# Valorization of Cocoa Pod Husk Through Integrated Drying and Probe Ultrasound-Assisted Extraction: Effects on Phenolic Composition, and Antioxidant and Cellular Anti-Inflammatory Activities

**DOI:** 10.3390/foods15152664

**Published:** 2026-07-29

**Authors:** Wachira Jirarattanarangsri, Thanyaporn Siriwoharn, Kongsak Boonyapranai, Rattana Muangrat

**Affiliations:** 1 Division of Food Science and Technology, Faculty of Agro-Industry, Chiang Mai University, Chiang Mai 50100, Thailand; wachira.j@cmu.ac.th (W.J.); thanyaporn.s@cmu.ac.th (T.S.); 2 Bioactive Compound Extraction Research Unit, Faculty of Agro-Industry, Chiang Mai University, Chiang Mai 50100, Thailand; 3 Research Institute for Health Sciences, Chiang Mai University, Chiang Mai 50200, Thailand; kongsak.b@cmu.ac.th; 4 Division of Food Process Engineering, Faculty of Agro-Industry, Chiang Mai University, Chiang Mai 50100, Thailand

**Keywords:** cocoa pod husk, probe ultrasound-assisted extraction, phenolic compounds, cellular anti-inflammatory activity, cytotoxicity

## Abstract

Cocoa pod husk, a major agro-industrial by-product of cocoa processing, represents an underutilized source of phenolic compounds with potential functional applications. This study developed an integrated extraction process combining drying pretreatments with probe ultrasound-assisted extraction to enhance the recovery and bioactivity of cocoa pod husk extracts. Preliminary solvent screening identified aqueous ethanol (1:3, *v*/*v*) as the most suitable extraction solvent, and a solid-to-solvent ratio of 1:9 (*w*/*v*) was subsequently selected for further extraction experiments. Under these extraction conditions, the type of drying pretreatment (tray drying or vacuum microwave drying), ultrasonic amplitude, and extraction time influenced the total phenolic content, phenolic composition, and antioxidant activities (DPPH, ABTS, and FRAP) of cocoa pod husk extracts. The evaluated extracts exhibited antibacterial activity against *Streptococcus mutans*. Cellular assays were performed using two selected extracts: Cocoa-A (vacuum microwave-dried) and Cocoa-B (tray-dried), which were prepared with aqueous ethanol (1:3, *v*/*v*) at a solid-to-solvent ratio of 1:9 (*w*/*v*) under 75% ultrasonic amplitude for 15 min. These extracts contained total phenolic contents of 5.41 ± 0.14 and 5.01 ± 0.14 mg GAE/g dried cocoa pod husk for Cocoa-A and Cocoa-B, respectively. Both extracts showed low cytotoxicity together with reduced intracellular reactive oxygen species and suppression of lipopolysaccharide-induced inflammatory mediators, including nitric oxide, IL-6, and TNF-α. The tray-dried extract (Cocoa-B) exhibited greater anti-inflammatory activity than the vacuum microwave-dried extract (Cocoa-A), indicating that the drying pretreatment influenced the biological activity of the extracts. This difference may also be associated with variations in phenolic composition, although the contribution of individual phenolic compounds was not investigated in the present study. These findings improve the understanding of how drying pretreatment and probe ultrasound-assisted extraction parameters influence the recovery of bioactive compounds from cocoa pod husk and provide a scientific basis for the further development of cocoa pod husk-derived bioactive ingredients for functional food, nutraceutical, and oral health-related applications.

## 1. Introduction

The cocoa industry generates large volumes of agro-industrial by-products, among which cocoa pod husk represents the most abundant fraction [1]. Although commonly discarded, cocoa pod husk has received increasing attention as a potential source of bioactive compounds and nutritional components, including dietary fiber, pectin, and phenolic compounds with antioxidant and biological activities [1,2,3,4]. These characteristics highlight cocoa pod husk as a promising low-cost raw material for sustainable valorization within circular bioeconomy systems [2]. Other cocoa residues, such as cocoa bean shell, have also been reported as sources of phenolic compounds, flavonoids, and methylxanthines with antioxidant and antimicrobial activities, further supporting the potential of cocoa-processing by-products as functional ingredient sources [5,6,7].

Drying pretreatment is an important step before extraction because it reduces moisture content, improves storage stability, and influences the physicochemical characteristics of plant matrices, which subsequently affect bioactive compound recovery [8,9,10,11]. Drying conditions can modify tissue structure, microstructural characteristics, and the stability of thermolabile and oxidation-sensitive compounds, thereby affecting extraction efficiency and biological functionality [12]. Conventional tray drying is widely used due to its simplicity and low cost; however, prolonged exposure to heat and oxygen may promote degradation of phenolic compounds [13]. In contrast, vacuum microwave drying enables rapid moisture removal through microwave volumetric heating under reduced pressure, which may minimize thermal and oxidative degradation while enhancing structural disruption and solvent accessibility during subsequent extraction [14,15,16,17]. Therefore, comparing different drying technologies provides insight into how drying-induced modifications influence the extractability and functionality of cocoa pod husk bioactive compounds.

The recovery of bioactive compounds is also strongly influenced by the extraction method. Conventional extraction techniques, such as maceration and Soxhlet extraction, generally require long processing times, high solvent consumption, and may cause degradation of sensitive compounds [18,19]. Probe ultrasound-assisted extraction has emerged as an efficient technique for bioactive compound recovery by promoting solvent penetration, cavitation, and mass transfer within plant matrices [20]. Compared with ultrasonic bath systems, probe ultrasonication provides more direct ultrasonic energy delivery, which can enhance extraction efficiency [20,21]. However, extraction performance depends strongly on operational parameters, particularly ultrasonic amplitude and extraction time. Inappropriate conditions may result in insufficient compound release, whereas excessive ultrasonic intensity or prolonged treatment may promote temperature increase and degradation of phenolic compounds [18,22]. Therefore, optimization of these parameters is necessary to maximize bioactive compound recovery while maintaining biological functionality.

Although cocoa pod husk has been investigated as a source of bioactive compounds, previous studies have mainly focused on extraction yield, total phenolic content, or antioxidant activity. Limited information is available regarding the integrated effects of drying pretreatment and probe ultrasound-assisted extraction on individual phenolic composition and cellular biological activities of cocoa pod husk extracts. In particular, the effects of combining different drying technologies with probe ultrasound-assisted extraction on antibacterial activity, cytotoxicity, intracellular reactive oxygen species (ROS) suppression, and anti-inflammatory responses remain insufficiently explored. Previous studies have reported the potential of drying pretreatments and probe ultrasound-assisted extraction for improving bioactive compound recovery from plant materials; however, the combined application of these approaches for cocoa pod husk extraction remains insufficiently explored.

Accordingly, this study aimed to evaluate the combined effects of tray drying and vacuum microwave drying followed by probe ultrasound-assisted extraction using an aqueous ethanol solvent system under different ultrasonic amplitudes and extraction times on the phenolic composition, antioxidant activity, antibacterial activity against *Streptococcus mutans*, cytotoxicity, and cellular biological activities of cocoa pod husk extracts. Aqueous ethanol was selected because ethanol and water are food-grade and low-toxicity solvents, and their combination provides suitable polarity for the recovery of a broad range of phenolic compounds compared with absolute ethanol or water alone. It was hypothesized that drying pretreatment and probe ultrasound-assisted extraction conditions would influence phenolic recovery and the associated antioxidant and cellular anti-inflammatory activities of cocoa pod husk extracts. To test this hypothesis, the extracts were characterized for their phenolic composition and evaluated for antioxidant, antibacterial, cytotoxic, intracellular reactive oxygen species (ROS)-suppressing, and anti-inflammatory activities. The findings provide insights into the effects of integrated drying pretreatment and probe ultrasound-assisted extraction on the recovery of bioactive compounds from cocoa pod husk and highlight its potential as a source of bioactive compounds for future applications.

## 2. Materials and Methods

### 2.1. Materials

#### 2.1.1. Raw Materials

Fresh cocoa pod husks were obtained from Kanwela Chocolate Co., Ltd. (Mueang Chiang Mai, Chiang Mai, Thailand).

#### 2.1.2. Chemicals and Reagents

Ethanol (AR grade) and methanol (AR grade) were purchased from RCI Labscan Ltd. (Bangkok, Thailand). Acetonitrile (HPLC grade), formic acid (HPLC grade), Folin–Ciocalteu reagent, sodium carbonate (AR grade), ferric chloride (AR grade), ferric sulfate (AR grade), sodium chloride (AR grade), sodium hydroxide (AR grade), sodium potassium tartrate (AR grade), and Griess reagent were purchased from Merck KGaA (Darmstadt, Germany). Gallic acid (analytical grade), 2,2-diphenyl-1-picrylhydrazyl (DPPH), 2,2′-azinobis (3-ethylbenzothiazoline-6-sulfonic acid) (ABTS), Trolox, and HPLC analytical standards, including gallic acid, protocatechuic acid, catechin, epicatechin, chlorogenic acid, caffeic acid, vanillic acid, *p*-coumaric acid, ferulic acid, rutin, quercetin, and kaempferol, were purchased from Sigma-Aldrich (St. Louis, MO, USA). Potassium persulfate (LR grade) was purchased from Ajax Finechem Pty Ltd. (Melbourne, Australia). 2,4,6-Tripyridyl-*s*-triazine (TPTZ) was purchased from Sigma-Aldrich (St. Louis, MO, USA).

#### 2.1.3. Bacterial Strain and Culture Media

*Streptococcus mutans* maintained by the Department of Medical Technology, Faculty of Associated Medical Sciences, Chiang Mai University, Chiang Mai, Thailand, was used as the test microorganism. Blood agar (BA), Tryptic Soy Agar (TSA), and Brain Heart Infusion (BHI) broth were purchased from Merck KGaA (Darmstadt, Germany).

#### 2.1.4. Cell Culture Reagents and Assay Kits

2,7-Dichlorofluorescein diacetate (DCFH-DA), dimethyl sulfoxide (DMSO), and Trolox were purchased from Sigma-Aldrich (St. Louis, MO, USA). Griess reagent, sodium chloride, sodium hydroxide, and sodium potassium tartrate were obtained from Merck (Darmstadt, Germany). MTT reagent was purchased from Thermo Fisher Scientific (Waltham, MA, USA). Mouse IL-6 and TNF-α ELISA kits were purchased from Abcam (Cambridge, UK).

### 2.2. Preparation of Cocoa Pod Husk and Selection of Solvent Systems for Probe Ultrasound-Assisted Extraction

Fresh cocoa pod husk was subjected to two different drying technologies, namely conventional tray drying and vacuum microwave drying, to evaluate the influence of drying pretreatment on phenolic recovery, antioxidant activity, and the biological functionality of extracts obtained by probe ultrasound-assisted extraction. Fresh cocoa pod husks were washed, sliced to an average thickness of approximately 2 mm to ensure uniform drying, and randomly divided into two groups for subsequent drying treatments.

For tray drying, approximately 3 kg of fresh cocoa pod husk was evenly distributed onto ten stainless-steel trays (50 cm × 90 cm × 4 cm; width × length × height; Navaloy Co., Ltd., Bang Khun Thian, Bangkok, Thailand) and dried in a hot-air tray dryer at 70 °C for 12 h until a final moisture content of approximately 4.4% (wet basis) was achieved. The drying process yielded approximately 0.42 kg of dried cocoa pod husk with a water activity (a_w_) of 0.486, as measured using a water activity meter (4TE Aqualab, Meter Group, Inc., Pullman, WA, USA).

Vacuum microwave drying was performed using a vacuum microwave dryer (Marchcool Industrial Co., Ltd., Chom Thong, Bangkok, Thailand) equipped with six magnetrons (800 W each), providing a total microwave power of 4800 W. Approximately 1 kg of fresh cocoa pod husk was loaded into a horizontally mounted perforated polypropylene (PP) rotating basket (15 rpm; bottom diameter 40 cm, top diameter 50 cm, height 65 cm). The basket was securely covered before being placed inside the vacuum microwave drying chamber. Drying was carried out under a vacuum pressure of −600 mmHg for 30 min until a final moisture content of approximately 4.4% (wet basis) was achieved. The drying process yielded approximately 0.15 kg of dried cocoa pod husk, which exhibited a water activity (a_w_) of 0.486 and a final moisture content of approximately 4.4% (wet basis).

Similar final moisture content and water activity (a_w_) values were achieved as practical drying endpoints to obtain comparable dried cocoa pod husk materials for subsequent extraction. Following drying, the cocoa pod husk was ground using a hammer mill (SK 300, Retsch GmbH, Haan, Germany) fitted with a 2-mm sieve. Particle size distribution was subsequently determined by sieve analysis using a Vibratory Sieve Shaker AS 200 control (Retsch GmbH, Haan, Germany) operated for 15 min at an amplitude of 2.0 mm with an interval time of 5 s. The ground powder exhibited a median particle size (D_50_) of approximately 421 µm. The same particle size distribution was maintained for all extraction experiments to ensure consistent extraction conditions, improve extraction uniformity, and minimize variation in solvent accessibility during probe ultrasound-assisted extraction. Preliminary solvent screening was performed to identify a suitable solvent system for phenolic recovery from cocoa pod husk. Distilled water, ethanol, and aqueous ethanol (1:3, *v*/*v*) were comparatively evaluated using an initial solid-to-solvent ratio of 1:7 (*w*/*v*) under fixed ultrasonication conditions. Tray-dried cocoa pod husk was selected as the representative material during the preliminary solvent screening to establish baseline extraction behavior prior to evaluating the effects of different drying pretreatments and probe ultrasound-assisted extraction conditions.

The investigated solvent systems were selected to represent different polarity conditions for evaluating their effects on phenolic recovery and antioxidant activity during probe ultrasound-assisted extraction. Water was selected as a highly polar solvent, ethanol as a lower-polarity food-grade solvent, and aqueous ethanol as an intermediate-polarity solvent system. A solid-to-solvent ratio of 1:7 (*w*/*v*) was used for the preliminary solvent screening to maintain consistent extraction conditions for comparing solvent performance. The most suitable solvent system identified from this preliminary screening was subsequently used for further extraction experiments, as described in Section 2.3.

### 2.3. Probe Ultrasound-Assisted Extraction of Bioactive Compounds from Cocoa Pod Husk

Probe ultrasonication-assisted extraction was performed using a VCX 500 ultrasonic processor (Sonics and Materials, Inc., Newtown, CT, USA) operating at a fixed frequency of 20 kHz. The ultrasonic probe had a diameter of 13 mm, and extraction was conducted in pulsed mode (5 s on/5 s off) to minimize excessive heat accumulation during sonication. The actual ultrasonic power applied during extraction was monitored from the ultrasonic processor output and varied depending on the applied amplitude. The measured ultrasonic power ranged from approximately 5 to 70 W, corresponding to a power density range of approximately 0.08–1.17 W/mL based on the extraction volume of 60 mL. The temperature of the extraction mixture was monitored throughout the sonication process without external temperature control, and the recorded temperature varied depending on ultrasonic amplitude and extraction time. Prior to extraction, dried cocoa pod husk samples obtained from Section 2.2 were ground into fine powder and transferred into 100 mL glass beakers.

Following the preliminary solvent screening described in Section 2.2, the extraction conditions were further evaluated by varying the solid-to-solvent ratio (1:5, 1:7, and 1:9, *w*/*v*) to determine its effect on phenolic composition. Based on these results, the most suitable solid-to-solvent ratio was selected for subsequent evaluation of ultrasonic amplitude (25%, 50%, and 75%) and extraction time (15, 30, and 45 min). Ultrasonic amplitude and extraction time were selected as the principal extraction variables because they are key operational parameters influencing solvent penetration, mass transfer, extraction efficiency, and the potential degradation of phenolic compounds during probe ultrasonication. The selected experimental ranges were based on previous studies reporting significant effects of these parameters on phenolic recovery during ultrasound-assisted extraction [23,24].

The solvent system identified from the preliminary screening was subsequently used to evaluate the effect of solid-to-solvent ratio. The selected solid-to-solvent ratio was then used to investigate the effects of ultrasonic amplitude and extraction time. The obtained extracts were analyzed for total phenolic content, phenolic composition, and antioxidant activities. Based on the extraction performance, selected extracts were further evaluated for antibacterial activity against *Streptococcus mutans*, cytotoxicity, intracellular reactive oxygen species (ROS) suppression, and anti-inflammatory activity.

### 2.4. Determination of Phenolic Compounds

Phenolic compounds were analyzed using a modified procedure based on Liaudanskas et al. [25]. Dried cocoa pod husk samples were first defatted prior to extraction. Briefly, 2 g of dried sample were mixed with 60 mL hexane and shaken overnight. The mixture was filtered through No. 4 filter paper, and the defatted residue was air-dried to remove residual solvent. The dried, defatted cocoa pod husk was then extracted with 99.99% ethanol at a ratio of 1 g sample to 10 mL solvent. The mixture was sonicated using an ultrasonic device for 15 min to enhance extraction efficiency and subsequently filtered to collect the liquid extract. The filtrate was mixed with 99.99% acetonitrile at a 1:1 (*v*/*v*) ratio and vortexed thoroughly to ensure homogeneity. The mixture was centrifuged at 13,000 rpm for 5 min, after which the clear supernatant was carefully collected and filtered through a 0.45 µm PTFE syringe filter. The filtered solution was transferred into HPLC vials for chromatographic analysis.

For cocoa extract samples, preparation involved direct dilution with 99.99% acetonitrile at a 1:1 (*v*/*v*) ratio, followed by vortex mixing and centrifugation at 13,000 rpm for 5 min. The resulting supernatant was filtered through a 0.45 µm PTFE syringe filter and transferred into HPLC vials prior to analysis.

Chromatographic separation of phenolic compounds was carried out using an HPLC system equipped with a C18-ODS column (5 µm particle size, 250 mm × 4.6 mm). The mobile phase consisted of solvent A (2% acetic acid in water), solvent B (100% acetonitrile), solvent C (deionized water), and solvent D (100% methanol). The system was operated at a flow rate of 1 mL/min with the column maintained at 30 °C. The injection volume was 10 µL. Detection was performed using a diode array detector at 280 nm with a total run time of 85 min. A 10% acetonitrile solution was used as the needle wash to minimize carryover. Phenolic standard solutions were prepared using the following reference compounds: gallic acid, theobromine, protocatechuic acid, *p*-hydroxybenzoic acid, catechin, chlorogenic acid, caffeine, vanillic acid, caffeic acid, syringic acid, epicatechin, vanillin, *p*-coumaric acid, ferulic acid, sinapic acid, rutin, myricetin, quercetin, and trans-cinnamic acid. Each compound was dissolved in an appropriate solvent to obtain working standard solutions, and all prepared standards were adjusted so that their final concentrations did not exceed 100 ppm prior to HPLC analysis. Phenolic compounds in the extracts were identified by comparing their retention times with those of the corresponding authentic standards. Quantification of individual phenolic compounds was performed by comparing the peak area of each compound in the sample with that of the corresponding reference standard of known concentration analyzed under identical chromatographic conditions.

### 2.5. Determination of Total Phenolic Content

The total phenolic content (TPC) of cocoa pod husk extracts was determined using the Folin–Ciocalteu colorimetric method according to Benítez-Correa et al. [26] with slight modifications. Briefly, 15 µL of extract solution was mixed with 2.0 mL of distilled water and 0.2 mL of Folin–Ciocalteu reagent. After incubation for 5 min at room temperature, 1.0 mL of 7.5% (*w*/*v*) sodium carbonate solution was added, and the mixture was incubated in the dark at room temperature for 1 h. The absorbance was measured at 765 nm using a UV–Vis spectrophotometer (Analytik Jena, Jena, Germany). A calibration curve was prepared using gallic acid as the standard at concentrations ranging from 0 to 500 mg/L. The TPC was calculated from the gallic acid calibration curve and expressed as milligrams of gallic acid equivalents per gram of dried cocoa pod husk (mg GAE/g dried cocoa pod husk). All analyses were performed in triplicate.

### 2.6. Determination of DPPH Radical Scavenging Activity

The DPPH radical scavenging activity was determined using the method described in a previous study [27] with slight modifications. A 1 mM DPPH solution was prepared in methanol. For the assay, 50 μL of the extract solution was mixed with 0.5 mL of distilled water and 0.5 mL of the DPPH solution. The mixture was vortex-mixed and incubated in the dark at room temperature for 30 min, after which the absorbance was measured at 515 nm using a UV–Vis spectrophotometer (Analytik Jena, Jena, Germany). Antioxidant activity was quantified using a Trolox calibration curve and expressed as micromoles of Trolox equivalent antioxidant capacity per gram of dried cocoa pod husk (μmol TEAC/g dried cocoa pod husk).

### 2.7. Determination of ABTS Radical Scavenging Activity

The ABTS radical scavenging activity was determined according to the modified method of Chen et al. [28]. The ABTS radical cation solution was prepared by mixing 7 mM ABTS solution with 2.45 mM potassium persulfate solution and incubating the mixture in the dark at room temperature for 12–16 h. Prior to analysis, the ABTS solution was diluted with ethanol to obtain an absorbance of 0.70 ± 0.05 at 734 nm. For the assay, 20 μL of the extract solution was diluted with 1 mL of distilled water, and 20 μL of the diluted sample was mixed with 1 mL of the ABTS radical solution. The mixture was vortex-mixed and incubated in the dark for 6 min, after which the absorbance was measured at 734 nm using a UV–Vis spectrophotometer (Analytik Jena, Jena, Germany). Antioxidant activity was calculated using a Trolox calibration curve (0–1000 μM) and expressed as micromoles of Trolox equivalent antioxidant capacity per gram of dried cocoa pod husk (μmol TEAC/g dried cocoa pod husk).

### 2.8. Determination of Ferric Reducing Antioxidant Power

The ferric reducing antioxidant power (FRAP) assay was performed according to the methods described by Benzie and Strain [29] and Szydłowska-Czerniaka et al. [30]. The FRAP reagent was freshly prepared by mixing 10 mL of 10 mM TPTZ solution (prepared in 40 mM HCl), 10 mL of 20 mM ferric chloride solution, and 100 mL of 0.3 M acetate buffer (pH 3.6). The reagent was incubated at 37 °C for 30 min prior to use. For the assay, 20 μL of the extract solution was diluted with 1 mL of distilled water, and 20 μL of the diluted sample was mixed with 1 mL of FRAP reagent. The mixture was vortex-mixed and incubated in the dark for 6 min. The absorbance was measured at 593 nm using a UV–Vis spectrophotometer (Analytik Jena, Jena, Germany). Antioxidant capacity was calculated using a Trolox calibration curve (0–1000 μM) and expressed as micromoles of Trolox equivalent antioxidant capacity per gram of dried cocoa pod husk (μmol TEAC/g dried cocoa pod husk).

### 2.9. Antibacterial Activity Assay by Disc Diffusion Method

The antibacterial activity of cocoa pod husk extracts against *Streptococcus mutans* was evaluated using the agar disc diffusion method. *Streptococcus mutans* was cultured in Brain Heart Infusion (BHI) broth at 37 °C for 24 h under 5% CO_2_. The bacterial suspension was adjusted with sterile 0.85% NaCl solution to the turbidity of a 0.5 McFarland standard, corresponding to approximately 1.5 × 10^8^ CFU/mL. A sterile cotton swab was used to evenly spread the bacterial suspension onto the surface of BHI agar plates.

Sterile paper discs (6 mm in diameter) impregnated with an appropriate volume of cocoa pod husk extract were placed on the inoculated agar surface. Chlorhexidine was used as the positive control, while the corresponding extraction solvent served as the negative control. The plates were incubated at 37 °C for 24 h under 5% CO_2_. After incubation, the diameters of the inhibition zones were measured in millimeters (mm). No inhibition zone was observed for the negative control. All experiments were performed in triplicate.

### 2.10. Minimum Bactericidal Concentration (MBC) Determination

The minimum bactericidal concentration (MBC) against Streptococcus mutans was determined using the broth macrodilution method. A fresh 24 h culture grown on blood agar was suspended in Brain Heart Infusion (BHI) broth and adjusted to a 0.5 McFarland standard (approximately 1 × 10^8^ CFU/mL), followed by dilution in BHI broth to obtain a final inoculum of approximately 1 × 10^6^ CFU/mL. The cocoa pod husk extracts were prepared in sterile distilled water and subjected to twofold serial dilutions in BHI broth to obtain the desired concentration range. Each dilution was inoculated with an equal volume of the bacterial suspension to obtain a final volume of 1 mL per tube. Control tubes included an extract control (without bacteria), a bacterial growth control, and a medium sterility control. The tubes were incubated at 35 ± 2 °C for 16–20 h under 5% CO_2_.

Following incubation, 0.01 mL aliquots from each test and control tube were subcultured onto tryptic soy agar plates using a calibrated loop and incubated at 35 ± 2 °C for 18–24 h under 5% CO_2_. MIC values were not reported because the turbidity of the cocoa pod husk extracts interfered with the visual determination of bacterial growth in the broth medium. Consequently, the antibacterial activity was evaluated based on MBC determination confirmed by subculture onto agar plates. The lack of MIC data is acknowledged as a limitation of the present study. The MBC was defined as the lowest extract concentration resulting in ≥99.9% bacterial killing, corresponding to no more than five colonies recovered from the 0.01 mL subculture.

### 2.11. Biological Activity Evaluation in Cultured Cells

Cocoa pod husk extracts obtained from the preliminary solvent screening and selected extraction conditions were subjected to solvent removal using a rotary evaporator. Ethanol extracts were concentrated under reduced pressure at 125 mbar and 50 °C, whereas water and aqueous ethanol extracts (ethanol:water = 1:3, *v*/*v*) were concentrated at 65 mbar and 50 °C until a constant weight was achieved and a concentrated extract was obtained. The obtained extracts were subsequently used for further biological analyses as described below.

#### 2.11.1. Intracellular Reactive Oxygen Species Inhibition in HepG2 Cells

The protective effect of cocoa pod husk extract against oxidative stress was evaluated in HepG2 cells by measuring intracellular reactive oxygen species (ROS) using the dichlorofluorescein diacetate (DCF-DA) assay. HepG2 cells were seeded in 6-well plates at an initial density of 4 × 10^3^ cells per well or in 96-well plates at 4 × 10^2^ cells per well and incubated at 37 °C in a CO_2_ incubator for 24 h. The culture medium was then replaced with 100 µL of fresh medium containing different concentrations of the extract (0–1000 µg/mL). The cells were subsequently co-incubated with hydrogen peroxide (H_2_O_2_) or lipopolysaccharide (LPS) for 24 h. After incubation, the cells were washed with phosphate-buffered saline (PBS) and treated with 25 µM 2,7-dichlorofluorescein diacetate (DCF-DA) for 30 min at 37 °C in the dark. Intracellular ROS production was determined by measuring the fluorescence intensity of DCF using a fluorescent microplate reader (Shimadzu, Kyoto, Japan) or flow cytometer at excitation and emission wavelengths of 488 and 540 nm, respectively. Trolox was used as a positive control (reference antioxidant), whereas untreated cells and H_2_O_2_-treated cells served as the negative and oxidative stress controls, respectively. Cell viability is expressed as a percentage relative to untreated control cells (100%). Data are presented as mean ± SD from three independent experiments.

#### 2.11.2. Cytotoxicity Assay in RAW 264.7 Macrophages

The cytotoxicity of the extract was evaluated in RAW 264.7 macrophage cells using the MTT assay. RAW 264.7 cells were seeded in 96-well plates at an initial density of 2.5 × 10^3^ cells per well and incubated at 37 °C in a CO_2_ incubator for 24 h. The cells were then treated with 100 µL of culture medium containing different concentrations of the extract (0–500 µg/mL) and incubated at 37 °C for 24 h. Cell viability was determined using MTT dye. After incubation with the reagent, the absorbance was measured using a microplate reader (BMG Labtech, Ortenberg, Germany). Cell viability was expressed as the percentage of surviving cells relative to the untreated control. All experiments were performed at least three times with three replicates for each treatment. Data are presented as mean ± standard deviation (SD) from three independent experiments; extract concentrations that maintained cell viability above 80% were considered non-cytotoxic and were selected for subsequent experiments.

#### 2.11.3. Determination of Nitric Oxide Production by Griess Reagent Assay

The inhibitory effect of the extract on nitric oxide (NO) production was evaluated in RAW 264.7 macrophages using the Griess reagent assay. RAW 264.7 cells were seeded in 96-well plates at a density of 2.5 × 10^4^ cells per well and incubated at 37 °C in a 5% CO_2_ incubator for 24 h. The cells were divided into three groups: untreated control cells cultured in medium alone, cells stimulated with lipopolysaccharide (LPS, 1 µg/mL), and cells treated with the extract at different concentrations followed by LPS stimulation. Dexamethasone was used as the positive control for the inhibition of nitric oxide production. The cells were pretreated with the extract for 2 h prior to LPS stimulation and incubated for an additional 24 h. After incubation, 100 µL of the culture supernatant was transferred to a 96-well plate and mixed with 100 µL of Griess reagent. The reaction mixture was incubated at room temperature in the dark for 15 min, and the absorbance was measured at 540 nm using a microplate reader (BMG Labtech, Ortenberg, Germany). Experiments were performed in triplicate, with at least three wells per treatment. Data are presented as mean ± standard deviation (SD) from three independent experiments. The highest non-cytotoxic extract concentration, along with two subsequent two-fold dilutions, was used for the assay.

#### 2.11.4. Determination of Interleukin-6 and Tumor Necrosis Factor-α Secretion in Lipopolysaccharide-Stimulated Cells Using Enzyme-Linked Immunosorbent Assay

Murine macrophage RAW264.7 cells were seeded in 6-well plates at an initial density of 1 × 10^6^ cells per well and incubated at 37 °C in a humidified atmosphere containing 5% CO_2_ for 24 h. After incubation, the cells were treated with culture medium containing the test compounds. The cells were divided into three groups. The control group was cultured with culture medium alone. The second group was treated with lipopolysaccharide (LPS) at a concentration of 1 µg/mL. The third group was treated with LPS in combination with different concentrations of the test compounds. Dexamethasone in combination with LPS was used as the positive control for the inhibition of inflammatory cytokine production. The cells were pretreated with dexamethasone or the test compounds for 2 h prior to LPS stimulation and then incubated with LPS for 24 h. After treatment, the culture supernatants were collected for the determination of interleukin-6 (IL-6) and tumor necrosis factor-α (TNF-α) secretion using an enzyme-linked immunosorbent assay (ELISA) according to the manufacturer’s instructions. The tested concentrations of the compounds were selected based on the highest non-cytotoxic concentration and were further prepared as two-fold serial dilutions to obtain three concentrations. All experiments were performed in triplicate, with at least three replicate wells per experiment. Data are presented as mean ± standard deviation (SD) from three independent experiments.

### 2.12. Statistical Analysis

All experiments were performed using three independent biological replicates, and each sample was analyzed in triplicate. Data were expressed as mean ± standard deviation (SD). Statistical differences among treatments were determined using one-way analysis of variance (ANOVA), followed by Duncans multiple range test at a significance level of *p* < 0.05. All statistical analyses were performed using SPSS software (version 17.0).

## 3. Results and Discussion

### 3.1. Phytochemical Profiles and Yields of Cocoa Pod Husk Extracts

A preliminary extraction study was conducted to evaluate the influence of solvent polarity and solid-to-solvent ratio on the recovery of phenolic compounds from cocoa pod husk under fixed probe ultrasonication conditions (50% amplitude, 30 min). Tray-dried cocoa pod husk was initially selected as the representative material to establish baseline extraction conditions before evaluating the effects of different drying pretreatments and probe ultrasound-assisted extraction parameters.

The initial profile of bioactive compounds in tray-dried cocoa pod husk before extraction was relatively limited, with epicatechin identified as the predominant detectable compound, whereas most phenolic acids, flavonoids, and methylxanthines were below the detection limit (Table 1). This observation suggests that a substantial proportion of phenolic compounds remained entrapped within the plant matrix or existed in bound forms with limited extractability in the untreated dried material.

Following probe ultrasonication-assisted extraction, a markedly broader phenolic profile was detected, particularly when aqueous ethanol (1:3, *v*/*v*) was used as the extraction solvent. The extracts contained substantially higher levels of epicatechin together with phenolic acids, including *p*-hydroxybenzoic acid, chlorogenic acid, syringic acid, and ferulic acid, as well as flavonoids such as rutin. Low levels of methylxanthines, including theobromine and caffeine, were also detected. The enhanced recovery of these compounds suggests that the applied probe ultrasound-assisted extraction conditions facilitated the extraction of phenolic compounds, possibly through improved solvent accessibility and mass transfer. Increasing the solid-to-solvent ratio from 1:5 to 1:9 (*w*/*v*) progressively enhanced phenolic recovery, with epicatechin remaining the dominant compound in all extracts. The improved extraction efficiency observed at higher solvent ratios may be attributed to increased solvent availability, which could facilitate mass transfer between the plant matrix and extraction medium during probe ultrasound-assisted extraction. Among the investigated conditions, aqueous ethanol (1:3, *v*/*v*) at a solid-to-solvent ratio of 1:9 (*w*/*v*) resulted in higher recovery of identified phenolic compounds compared with other solid-to-solvent ratios (Table 1).

In contrast, extraction using absolute ethanol resulted in a substantially narrower phenolic profile and lower extraction efficiency. Only a limited number of compounds, primarily epicatechin together with small amounts of ferulic acid and rutin, were detected. This behavior may be attributed to the relatively lower polarity of absolute ethanol, which limited extraction of more polar phenolic constituents from cocoa pod husk. Conversely, extraction using water alone produced highly viscous gel-like extracts, likely because cocoa pod husk contains substantial amounts of water-soluble pectic polysaccharides that readily dissolve during aqueous extraction [31]. The co-extraction of pectin increases extract viscosity and promotes gel formation, which may hinder solvent diffusion, reduce mass transfer of phenolic compounds, and interfere with subsequent analytical measurements. Previous studies have also reported that pectin is one of the major structural polysaccharides in cocoa pod husk and that its extraction is enhanced under aqueous extraction conditions, particularly when assisted by cell wall disruption techniques such as ultrasound [31,32]. Consequently, the increased viscosity of the aqueous extracts may have limited the efficient recovery and subsequent determination of phenolic compounds despite the high polarity of water.

The differences in individual phenolic profiles observed in Table 1 may be associated with the chemical structures and solubility characteristics of each compound in the applied extraction system. The aqueous ethanol solvent used in this study provided suitable polarity for the recovery of various polar phenolic compounds, particularly phenolic acids and polar flavonoid compounds. For example, rutin was detected among the flavonoid compounds identified in cocoa pod husk extracts, which may be attributed to the presence of glycosyl moieties that increase molecular polarity and enhance its solubility in aqueous ethanol systems [33,34]. In contrast, quercetin was not detected under the applied extraction and analytical conditions. This observation may be associated with the relatively lower polarity and aqueous solubility of quercetin aglycone compared with its glycosylated derivatives. In addition, the possible presence of quercetin derivatives in conjugated forms within the cocoa pod husk matrix and the absence of an acid hydrolysis step prior to analysis may have contributed to the non-detection of free quercetin aglycone [33,35]; however, these factors were not investigated in the present study. Therefore, the selected aqueous ethanol extraction system may have been more favorable for the recovery of polar phenolic compounds and flavonoid derivatives than less polar free flavonol aglycones. Previous studies have reported that the recovery of flavonoid aglycones may be improved by optimizing solvent polarity or incorporating acid or enzymatic hydrolysis to cleave glycosidic linkages before extraction or analysis [33,34]. The observed variations among individual phenolic compounds may therefore reflect differences in molecular polarity, structural characteristics, and interactions with the cocoa pod husk matrix. Further studies focusing on the targeted recovery of flavonoid aglycones should evaluate alternative solvent systems and hydrolysis-assisted extraction strategies to improve extraction efficiency.

Overall, aqueous ethanol (1:3, *v*/*v*) was identified as a suitable extraction solvent for recovering phenolic compounds with diverse polarities, showing higher extraction performance than absolute ethanol or water alone during probe ultrasound-assisted extraction. Based on the evaluated solid-to-solvent ratios, aqueous ethanol at a ratio of 1:9 (*w*/*v*) provided favorable phenolic compound recovery and was therefore selected for subsequent extraction studies. The effects of drying pretreatment and probe ultrasonication conditions on phenolic recovery and antioxidant activity of cocoa pod husk extracts are presented in Table 2, Table 3 and Table 4. Under comparable extraction conditions, vacuum microwave-dried (MD) cocoa pod husk generally showed higher total phenolic content and antioxidant activity than tray-dried (TD) samples. However, the differences varied with ultrasonic amplitude and extraction time, suggesting that drying pretreatment affected the accessibility and recovery of bioactive compounds during subsequent probe ultrasound-assisted extraction.

Vacuum microwave drying may enhance phenolic extractability through rapid volumetric heating and internal moisture vaporization, which could promote structural modification and improve the accessibility of intracellular compounds within the cocoa pod husk matrix [36,37]. The generation of internal steam may create microfractures and increase tissue porosity, thereby facilitating solvent penetration and improving mass transfer during subsequent probe ultrasound-assisted extraction [36,37]. In addition, the reduced oxygen environment during vacuum microwave drying may limit oxidative degradation of oxidation-sensitive phenolic compounds [38]. These effects may contribute to the enhanced recovery of phenolic compounds and the increased antioxidant activity observed in microwave-dried samples after ultrasound-assisted extraction. In contrast, tray drying involves prolonged thermal exposure under atmospheric conditions, which may promote partial oxidation and reduce the extractability of certain phenolic constituents [39]. Similar trends have been reported in microwave-dried plant materials, where reduced thermal degradation and improved compound accessibility enhanced the recovery of bioactive compounds compared with conventional hot-air drying systems [15,40,41,42].

While vacuum microwave drying improved the accessibility of bioactive compounds prior to extraction, phenolic recovery was also influenced by probe ultrasound-assisted extraction conditions. At shorter extraction times, particularly at low to moderate ultrasonic amplitudes (25–50%), 15 min extraction generally resulted in lower phenolic recovery, likely due to insufficient sonication time for effective solvent penetration and mass transfer. However, when a higher amplitude (75%) was applied, comparable or even higher phenolic recovery was achieved within 15 min, suggesting that higher ultrasonic amplitude may enhance the disruption of the plant matrix and facilitate the release of phenolic compounds within a shorter extraction period. Increasing the extraction time from 15 to 30 min improved phenolic recovery under selected ultrasonication conditions, particularly at 50% amplitude, whereas further extension or higher amplitude did not consistently enhance extraction. However, prolonged sonication (45 min) did not further improve recovery and, under some conditions, resulted in lower phenolic content and DPPH activity, possibly due to degradation of oxidation-sensitive compounds during extended processing. Similar decreases in phenolic stability under excessive sonication conditions have been reported in plant extraction systems [43,44]. Although vacuum microwave drying showed potential for improving bioactive compound recovery, drying efficiency, energy consumption, and processing cost were not evaluated in this study and should be considered in future techno-economic assessments.

Ultrasonic amplitude further affected extraction performance. At 25% amplitude, phenolic recovery and antioxidant activity were comparatively lower, possibly due to insufficient ultrasonic energy input for effective solvent–matrix interactions and mass transfer. Increasing the amplitude to 50% substantially improved extraction efficiency, suggesting that this condition enhanced solvent accessibility and promoted the recovery of phenolic compounds. Under these conditions, increasing the amplitude to 50% generally improved extraction performance for both MD and TD samples, particularly antioxidant activity, whereas the highest total phenolic contents were observed under selected 75% amplitude conditions. This finding suggests that higher amplitude may enhance the release of phenolic compounds from the matrix.

Interestingly, extraction at 75% amplitude demonstrated different effects depending on extraction duration. At a short extraction time (15 min), the high amplitude condition resulted in phenolic recovery and antioxidant activity comparable to, or in some cases higher than, those obtained at 50% amplitude for 30 min. This finding suggests that a higher ultrasonic amplitude applied for a shorter duration may enhance extraction efficiency within a limited processing period. However, when high amplitude was combined with prolonged extraction time (30–45 min), phenolic recovery and antioxidant activity decreased. This decline may be associated with extended ultrasound exposure, which could promote the degradation of oxidation-sensitive phenolic compounds during prolonged processing, as reported in previous studies [45,46].

These findings demonstrated that phenolic recovery from cocoa pod husk was influenced by both ultrasonic amplitude and extraction time, as well as the potential degradation of phenolic compounds during prolonged ultrasound processing. Among the evaluated extraction conditions, 75% amplitude for 15 min and 50% amplitude for 30 min showed comparatively high phenolic recovery and antioxidant activity. Both conditions resulted in efficient phenolic recovery under different ultrasonic amplitude and extraction time conditions. The 75% amplitude for 15 min condition achieved high phenolic recovery within a shorter extraction period, whereas the 50% amplitude for 30 min condition also provided high phenolic recovery with a longer extraction duration. These observations highlight that appropriate ultrasonic amplitude and extraction duration are required to enhance phenolic recovery, as excessive increases in a single extraction parameter may not necessarily improve extraction performance. Similar extraction behaviors have been reported in other ultrasound-assisted extraction systems involving phenolic compounds from plant matrices [47,48,49].

As shown in Table 2, antioxidant activities determined by DPPH, ABTS, and FRAP assays showed varying trends among extracts obtained under different ultrasonication conditions. Although some extracts with higher total phenolic content exhibited relatively high antioxidant activities, the relationship was not directly proportional across all samples. For example, the extracts with the highest total phenolic content did not always show the highest antioxidant activity values. These differences indicate that antioxidant functionality was not determined solely by total phenolic content, but was also influenced by individual phenolic composition, structural characteristics, and possible interactions among extract constituents. DPPH and ABTS assays primarily evaluate radical scavenging activity, whereas FRAP reflects ferric ion reducing capacity. Therefore, variations in antioxidant responses among extracts may be attributed to differences in the types and relative abundance of bioactive compounds recovered under different drying pretreatments and ultrasound-assisted extraction conditions.

Individual phenolic profiling, as shown in Table 3 and Table 4, further demonstrated that drying pretreatment and probe ultrasound-assisted extraction conditions influenced the phenolic composition of cocoa pod husk extracts. The concentrations of individual phenolic compounds varied among samples subjected to different drying pretreatments and extraction conditions, indicating that the response of individual phenolic constituents was compound-dependent. Although some phenolic compounds showed higher concentrations under selected vacuum microwave-drying conditions, these improvements were not observed consistently for all compounds or extraction conditions. In contrast, several compounds showed comparable or higher concentrations in tray-dried samples, suggesting that drying pretreatment influenced the phenolic profile differently depending on the individual compound. For example, myricetin concentrations were higher in several tray-dried extracts, highlighting that the effect of drying pretreatment on phenolic composition cannot be explained solely by total phenolic recovery. Overall, the differences observed in individual phenolic profiles suggest that the choice of drying pretreatment and ultrasound-assisted extraction conditions influenced the composition of extracted phenolic compounds, rather than uniformly increasing the concentration of all phenolic constituents.

Overall, the combined results demonstrate that drying pretreatment and probe ultrasound-assisted extraction influenced the recovery of phenolic compounds and the antioxidant functionality of cocoa pod husk extracts. Based on previous reports, vacuum microwave drying may modify the plant matrix through rapid internal heating and moisture removal, which could facilitate solvent penetration during subsequent probe ultrasound-assisted extraction. Ultrasonic cavitation generates localized shear forces and microjets that disrupt plant cell walls and promote the release of intracellular phenolic compounds. Therefore, differences in the physicochemical characteristics of the cocoa pod husk matrix resulting from the drying pretreatments may have influenced cavitation efficiency and the extraction of individual phenolic compounds. These effects may explain the differences observed in total phenolic content, HPLC phenolic profiles, and antioxidant activities among the extracts. However, changes in the plant microstructure and cavitation behavior were not evaluated in the present study. Collectively, these findings highlight the importance of appropriate drying pretreatment and probe ultrasound-assisted extraction for improving the recovery of bioactive compounds from cocoa pod husk and support its potential valorization as a sustainable source of functional ingredients for food and health-related applications.

### 3.2. Antibacterial and Bactericidal Activity Against Streptococcus Mutans

The antibacterial activity of cocoa pod husk extracts was evaluated *against Streptococcus mutans* to explore the potential utilization of cocoa by-product extracts in oral health-related applications. Probe ultrasonication-assisted extraction using ethanol and aqueous ethanol enabled recovery of phenolic compounds together with low levels of methylxanthines, particularly theobromine, as presented in Table 1. Previous studies have reported that cocoa-derived phenolics and methylxanthines may contribute to antibacterial activity against oral pathogens through multiple mechanisms, including membrane disruption and interference with bacterial metabolism [8,9]. Therefore, cocoa pod husk extracts obtained under different solvent conditions were selected for antibacterial evaluation.

**Figure 1:**
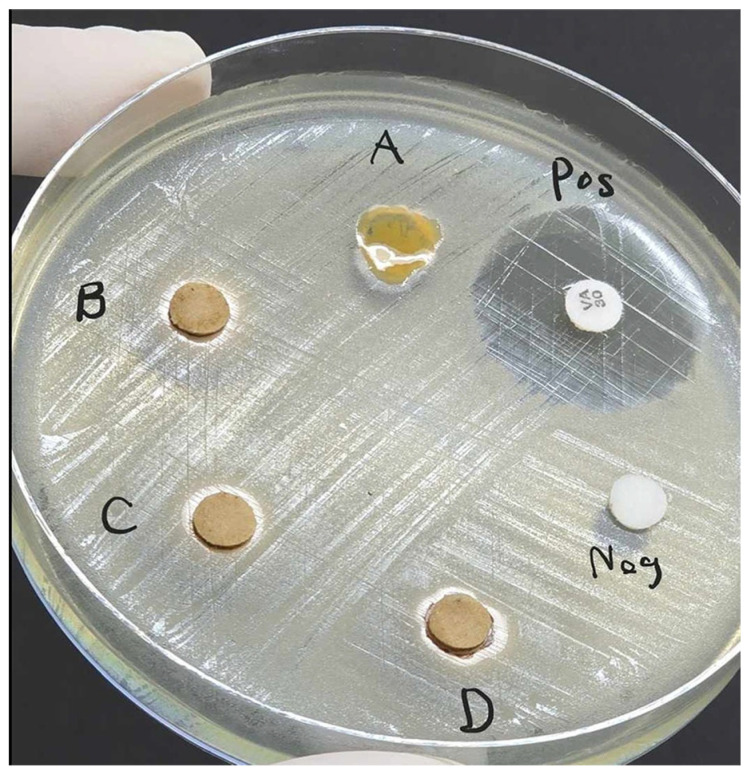
Inhibition of *Streptococcus mutans* by tray-dried cocoa pod husk extracts determined using the disc diffusion method. The extracts were obtained using ethanol (solid-to-solvent ratio of 1:7) and aqueous ethanol (1:3 *v*/*v*) at solid-to-solvent ratios of 1:5, 1:7, and 1:9 (*w*/*v*), followed by probe ultrasound-assisted extraction at an amplitude of 50% for 30 min. **A**: ethanol extract at a solid-to-solvent ratio of 1:7; **B**: aqueous ethanol extract (1:3, *v*/*v*) at a solid-to-solvent ratio of 1:9; **C**: aqueous ethanol extract (1:3, *v*/*v*) at a solid-to-solvent ratio of 1:7; **D**: aqueous ethanol extract (1:3, *v*/*v*) at a solid-to-solvent ratio of 1:5. Chlorhexidine was used as the positive control, and the extraction solvent was used as the negative control.

The antibacterial activity of tray-dried cocoa pod husk extracts was initially assessed using the disc diffusion method. The extracts were prepared under probe ultrasonication at an amplitude of 50% for 30 min using different solvent systems and solid-to-solvent ratios. Extract A was the ethanol extract prepared at a solid-to-solvent ratio of 1:7 (*w*/*v*), whereas extracts B, C, and D were aqueous ethanol extracts (1:3, *v*/*v*) prepared at solid-to-solvent ratios of 1:9, 1:7, and 1:5 (*w*/*v*), respectively. As shown in Figure 1, all cocoa pod husk extracts exhibited inhibitory activity against *Streptococcus mutans*, as evidenced by the formation of clear inhibition zones surrounding the discs. The inhibition-zone diameters of extracts A and D were 8.5 ± 0.03 and 8.5 ± 0.05 mm, respectively, whereas those of extracts B and C were 6.5 ± 0.01 and 6.5 ± 0.00 mm, respectively. Although these inhibition zones were substantially smaller than the inhibition zone produced by chlorhexidine (29.5 ± 0.03 mm), all extracts produced measurable inhibition zones, indicating weak antibacterial activity under the conditions tested. No inhibition was observed for the corresponding solvent controls.

The relatively comparable inhibition zones among the extracts suggest that probe ultrasonication effectively facilitated the recovery of antibacterial constituents from cocoa pod husk under the investigated extraction conditions. In particular, aqueous ethanol enabled extraction of phenolic compounds with broader polarity ranges compared with absolute ethanol, resulting in comparable or slightly enhanced antibacterial performance. These findings indicate that solvent polarity and probe ultrasound-assisted extraction conditions affected the recovery of multifunctional bioactive compounds, which may contribute to the observed antibacterial functionality.

Although phenolic compounds and methylxanthines, including theobromine, were detected in the cocoa pod husk extracts, the present study did not evaluate the contribution of individual compounds to the observed antibacterial activity. Therefore, the specific constituents responsible for the antibacterial effects could not be identified. Previous studies have reported that phenolic acids and flavonoids exert antibacterial effects through membrane disruption, alteration of cellular permeability, interference with enzymatic systems, and inhibition of microbial metabolism, whereas methylxanthines such as theobromine may additionally contribute to anti-cariogenic activity against *Streptococcus mutans* [8,9]. Although differences in antibacterial activity were observed among the aqueous ethanol and ethanol extracts, the present study did not determine whether these variations were associated with specific phenolic compounds, methylxanthines, or other extractable constituents.

Nevertheless, the present study focused primarily on evaluating the overall antibacterial potential of cocoa pod husk extracts rather than determining the contribution of individual compounds to the observed antibacterial activity. Therefore, the relative roles and possible synergistic interactions among phenolics, methylxanthines, and other extractable constituents could not be fully elucidated. Further studies involving compound isolation, fractionation, and mechanistic evaluation are required to identify the principal antibacterial components and to better understand the mechanisms underlying the inhibitory activity of cocoa pod husk extracts against *Streptococcus mutans*.

Overall, the findings demonstrate that cocoa pod husk, an abundant cocoa-processing by-product, represents a potential source of bioactive compounds with antibacterial activity against *Streptococcus mutans*. The integration of an aqueous ethanol solvent systems with probe ultrasound-assisted extraction provides a promising approach for recovering bioactive compounds from cocoa pod husk. However, further studies involving purification or enrichment of the active constituents, together with additional biological evaluation, are required before considering practical applications in oral health-related or functional food formulations.

Following the disc diffusion assay, the bactericidal activity of the tray-dried cocoa pod husk extracts against *Streptococcus mutans* was further evaluated by determining the minimum bactericidal concentration (MBC). Consistent with the disc diffusion results, extract A exhibited the lowest MBC value (417.5 mg/mL), whereas extracts B, C, and D exhibited MBC values greater than 1250 mg/mL, indicating limited bactericidal activity within the tested concentration range.

The differences in bactericidal activity among the tray-dried cocoa pod husk extracts suggest that the extraction solvent influenced the recovery of antibacterial constituents during probe ultrasound-assisted extraction. Although the aqueous ethanol extracts generally yielded higher concentrations of several phenolic compounds, the ethanol extract (extract A) exhibited the greatest bactericidal activity, with the lowest MBC value. This finding indicates that greater phenolic recovery did not necessarily correspond to stronger bactericidal activity against *Streptococcus mutans*, suggesting that the antibacterial effect may depend on the composition and relative abundance of bioactive constituents rather than their total phenolic content alone. Notably, extracts A and D produced comparable inhibition-zone diameters in the disc diffusion assay but differed markedly in their MBC values. This discrepancy may be attributed to the different principles underlying the two assays. The disc diffusion method is influenced by both the antibacterial activity of the extract and the diffusion of its constituents through the agar matrix, whereas the MBC assay directly evaluates bactericidal activity in a liquid medium. Consequently, similar inhibition-zone diameters do not necessarily indicate equivalent bactericidal activity. However, because the extracts were not fractionated or chemically characterized beyond the quantified compounds, the specific constituents responsible for the observed bactericidal effects could not be conclusively identified in the present study.

Consistent with the antibacterial activity described above, the relatively high MBC values indicate that substantially higher extract concentrations were required to achieve bactericidal effects against *Streptococcus mutans*. Nguyen and Nguyen [50] reported that cocoa pod husk naturally contains relatively low levels of theobromine (approximately 6.79 mg/100 g dry weight), which may limit the abundance of this bioactive constituent in crude extracts. In agreement with this observation, the present study detected relatively low concentrations of theobromine (0.92–4.18 µg/g dried cocoa pod husk; Table 1), suggesting that the observed bactericidal activity may be associated with the limited concentration of antibacterial constituents within the crude extract matrix. Similarly to other crude plant-derived extracts, the antibacterial effects observed in this study may result from the combined actions of multiple phytochemicals rather than a single highly potent antibacterial compound. However, because the extracts were evaluated as crude preparations without purification, fractionation, or enrichment of active constituents, the specific contributions of individual compounds and possible synergistic interactions among bioactive constituents could not be determined. Further purification, fractionation, or enrichment strategies may enhance antibacterial potency and provide a better understanding of the compounds responsible for the observed activity. Nevertheless, the relatively high MBC values suggest that direct application of the crude extracts at bactericidal concentrations may be limited, and further studies are required to evaluate their efficacy after purification or enrichment and to investigate the underlying antibacterial mechanisms before considering potential applications in oral health-related formulations.

It should be noted that MIC values were not determined in the present study because the inherent turbidity of the cocoa pod husk extracts interfered with the visual assessment of bacterial growth in the broth medium. Therefore, antibacterial activity was evaluated based on MBC values determined by subculturing onto agar plates. In addition, the disc diffusion assay was used to assess antibacterial activity, and inhibition-zone diameters were quantitatively measured. The absence of MIC data is acknowledged as a limitation of the present study.

### 3.3. In Vitro Cytotoxicity and Intracellular ROS Suppression in HepG2 Cells

Evaluation of antioxidant and anti-inflammatory activities of cocoa extracts in cell-based models is important to determine their biological efficacy and safety in living systems. Compared with chemical assays, cell-based assays provide more physiologically relevant evidence by assessing the ability of bioactive compounds to reduce oxidative stress and regulate inflammatory responses in cells, thereby supporting their potential health benefits and applications in functional foods or nutraceuticals. Cocoa pod husk extracts from vacuum microwave-dried and tray-dried samples were prepared using probe ultrasonication with aqueous ethanol (1:3, *v*/*v*) at a solid-to-solvent ratio of 1:9 (*w*/*v*). Extraction at 75% amplitude for 15 min resulted in the highest total phenolic content (Table 3 and Table 4), with values of 5.01 ± 0.14 and 5.41 ± 0.14 mg GAE/g dried cocoa pod husk for the tray-dried extract (Cocoa-B) and vacuum microwave-dried extract (Cocoa-A), respectively. The extract obtained from vacuum microwave-dried cocoa pod husk was designated as Cocoa-A, whereas the extract obtained from tray-dried cocoa pod husk was designated as Cocoa-B. These extracts were subsequently evaluated for cytotoxicity and antioxidant activity in HepG2 cells, as well as for cytotoxicity and anti-inflammatory activity in lipopolysaccharide (LPS)-stimulated RAW264.7 macrophages. Cell viability was initially evaluated over a concentration range of 0–1000 µg/mL to establish an appropriate concentration range for subsequent biological assays. Based on the MTT assay, concentrations up to 300 µg/mL maintained cell viability above 80%; therefore, only concentrations of 50–300 µg/mL were selected for the subsequent anti-inflammatory assays.

**Figure 2:**
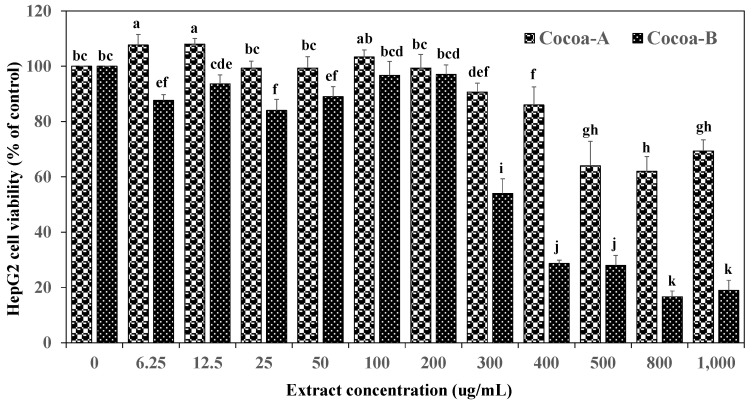
Cytotoxic effects of dried cocoa pod husk extracts (Cocoa-A and Cocoa-B) on HepG2 cells. HepG2 cells were treated with extracts (0–1000 µg/mL) for 24 h, and cell viability was measured by the MTT assay. Cocoa-A and Cocoa-B represent extracts from vacuum microwave-dried and tray-dried cocoa pod husk, respectively. Different lowercase letters above the bars indicate significant differences among treatments (*p* < 0.05).

Figure 2 illustrates the cytotoxic effects of Cocoa-A and Cocoa-B extracts on HepG2 cells after 24 h of treatment at concentrations ranging from 0 to 1000 µg/mL, as determined by the MTT assay. At low concentrations (6.25–200 µg/mL), Cocoa-A maintained cell viability close to or slightly above 100%, indicating negligible cytotoxicity and suggesting a mild stimulatory effect on cellular metabolic activity. In contrast, Cocoa-B exhibited slightly lower cell viability within the same concentration range (approximately 84–97%), although the values remained above the commonly accepted non-cytotoxic threshold of 80%.

A clear concentration-dependent reduction in cell viability was observed at higher concentrations for both extracts. However, the decline was markedly more pronounced for Cocoa-B. At 300 µg/mL, cell viability decreased sharply to approximately 54% for Cocoa-B, whereas Cocoa-A maintained a relatively higher viability of about 91%. This difference became even more evident at concentrations of 400–1000 µg/mL, where Cocoa-B reduced cell viability to below 30%, while Cocoa-A showed a more moderate reduction.

The greater cytotoxicity observed for Cocoa-B may be associated with differences in chemical composition arising from the drying process of the cocoa pod husk. Vacuum microwave drying can enhance the extraction of certain bioactive compounds, including phenolic compounds and other secondary metabolites, which may exert stronger biological activity. While these compounds contribute to antioxidant and anti-inflammatory effects, higher concentrations may also interfere with cellular metabolism or induce oxidative stress-related responses, leading to reduced cell viability. In contrast, Cocoa-A, derived from tray-dried material, likely contains lower concentrations or different profiles of these bioactive constituents, resulting in milder cytotoxic effects.

The difference in cell viability between Cocoa-A and Cocoa-B may be associated with changes in the chemical composition of the extracts caused by the drying processes. Drying conditions are known to significantly influence the retention of phenolic and other bioactive compounds in plant materials. Microwave-vacuum drying generally preserves higher levels of phenolic and flavonoid compounds because of the shorter processing time and reduced oxygen exposure, which limit oxidative degradation of these compounds [14,51]. In contrast, conventional hot-air drying methods such as tray drying involve longer thermal exposure, which may promote oxidation and thermal degradation of polyphenols and reduce antioxidant activity [52,53]. Such changes in the chemical composition of the extracts could influence the cellular responses observed in the cytotoxicity assay using HepG2 cells, resulting in slightly lower cell viability for the extract obtained from tray-dried samples.

Overall, the results demonstrate that both extracts are relatively safe at low to moderate concentrations, while higher concentrations, particularly of Cocoa-B, significantly decrease HepG2 cell viability. These findings highlight the importance of selecting appropriate concentration ranges for subsequent cellular assays and suggest that differences in processing methods can influence the biological activity of cocoa pod husk extracts.

**Figure 3:**
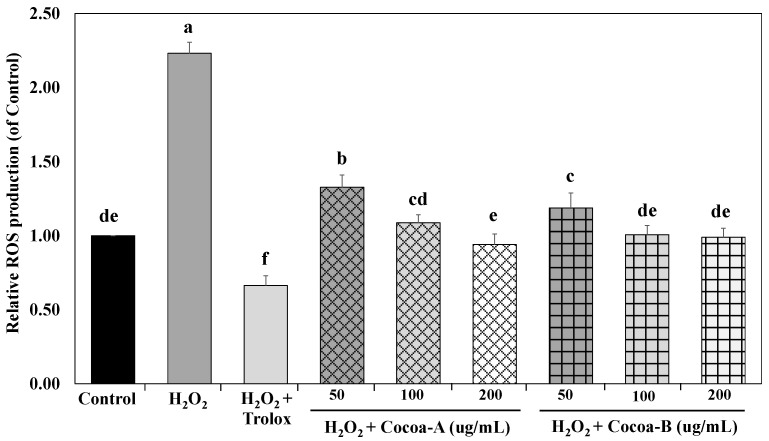
Antioxidant activity of Cocoa-A and Cocoa-B extracts in HepG2 cells. Intracellular ROS levels were determined using the DCF assay after 24 h of treatment with the extracts (50, 100, and 200 µg/mL). Results are expressed as percentage viability relative to untreated control cells. Different lowercase letters above the bars indicate significant differences among treatments (*p* < 0.05).

Figure 3 presents the intracellular antioxidant activity of Cocoa-A and Cocoa-B extracts in HepG2 cells subjected to oxidative stress induced by hydrogen peroxide (H_2_O_2_), as evaluated using the DCF assay. In the untreated control group, the relative intracellular reactive oxygen species (ROS) level remained close to the baseline value of approximately 1.0. Exposure to H_2_O_2_ markedly increased ROS production to approximately 2.2-fold of the control level, indicating successful induction of oxidative stress in HepG2 cells. Treatment with Trolox, used as a reference antioxidant, substantially decreased ROS levels to approximately 0.66, confirming the sensitivity and reliability of the assay system for detecting intracellular oxidative stress.

Hydrogen peroxide (H_2_O_2_) is widely used to induce intracellular oxidative stress through ROS generation in cell-based antioxidant assays [54,55,56]. Both Cocoa-A and Cocoa-B extracts significantly suppressed H_2_O_2_-induced ROS generation in a concentration-dependent manner. For Cocoa-A, ROS levels decreased from approximately 1.33 at 50 µg/mL to 1.09 and 0.94 at 100 and 200 µg/mL, respectively. A similar pattern was observed for Cocoa-B, where ROS levels declined from about 1.19 at 50 µg/mL to 1.01 and 0.99 at 100 and 200 µg/mL, respectively. At the highest concentration tested, both extracts reduced ROS levels to values approaching the basal level observed in untreated cells, indicating strong protection against oxidative stress.

Cocoa pod husk contains phenolic compounds that have been reported to possess antioxidant properties [57,58]. The comparable ROS-scavenging activities observed for Cocoa-A and Cocoa-B suggest that both drying methods preserved the antioxidant potential of the extracts. The concentration-dependent reduction in intracellular ROS indicates that increasing extract concentration enhanced the antioxidant response in HepG2 cells. However, the molecular mechanisms underlying ROS modulation were not investigated in the present study.

Overall, both Cocoa-A and Cocoa-B extracts effectively reduced intracellular ROS accumulation in H_2_O_2_-induced HepG2 cells, indicating their ability to protect hepatocyte-like cells from oxidative stress. These findings highlight the potential of cocoa pod husk, an abundant agricultural by-product, as a promising source of natural antioxidant compounds with possible applications in functional foods or nutraceutical formulations.

### 3.4. In Vitro Anti-Inflammatory Responses in LPS-Stimulated RAW264.7 Macrophages

**Figure 4:**
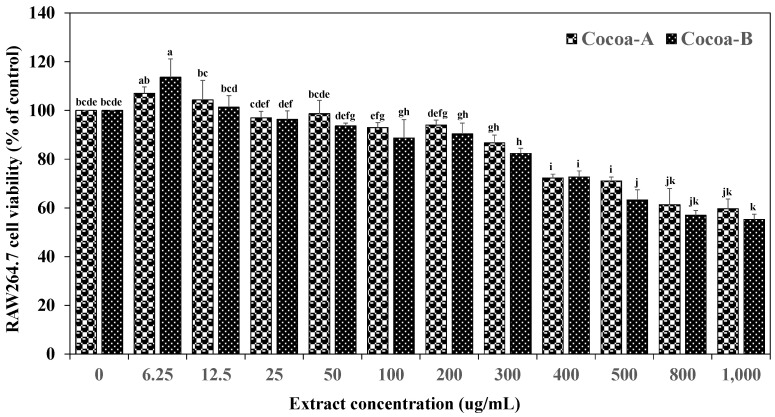
Cytotoxic effects of Cocoa-A and Cocoa-B extracts on RAW264.7 cells. Cell viability was assessed using the MTT assay after 24 h of treatment with the extracts (0–1000 µg/mL). Results are expressed as percentage viability relative to untreated control cells (100%). Different lowercase letters above the bars indicate significant differences among treatments (*p* < 0.05).

Figure 4 illustrates the effects of Cocoa-A and Cocoa-B extracts on the viability of RAW264.7 macrophage cells after 24 h of exposure, as evaluated using the MTT assay. Cell viability was expressed as a percentage relative to untreated control cells (100%). Overall, both cocoa pod husk extracts exhibited relatively low cytotoxicity over a wide concentration range, indicating good cellular compatibility with macrophage cells at moderate concentrations.

At lower concentrations (6.25–200 µg/mL), both extracts maintained high cell viability, generally exceeding 90%, indicating low cytotoxicity toward RAW264.7 macrophages within this range. Cell viability values slightly exceeding 100% were observed at the lowest extract concentration. However, the present study did not investigate the underlying cause of this observation, and therefore no mechanistic explanation can be established. Further studies are required to clarify whether this response reflects biological variability, assay-related effects, or changes in cellular metabolic activity.

As the concentration increased beyond 300 µg/mL, a gradual decline in cell viability was observed for both extracts, indicating a concentration-dependent effect on macrophage metabolic activity. At 500 µg/mL, cell viability decreased to approximately 70% for Cocoa-A and about 63% for Cocoa-B, while further reductions were observed at 800–1000 µg/mL. Because the MTT assay measures mitochondrial dehydrogenase activity, decreases in the measured signal may reflect reduced mitochondrial metabolic activity rather than direct cell death [59]. The reduction in MTT signal at higher concentrations indicates reduced metabolic activity under the experimental conditions.

Although both extracts exhibited similar cytotoxicity profiles, Cocoa-B showed slightly lower viability values at higher concentrations (≥500 µg/mL). This difference may be associated with variations in phytochemical composition arising from the drying process applied prior to extraction. Drying techniques influence the stability and extractability of phenolic compounds in plant matrices. In particular, microwave-assisted drying under vacuum conditions can promote rapid internal heating and structural disruption of plant tissues, facilitating the release of phenolic compounds that are otherwise bound within the cell wall matrix [14,60]. As a result, extracts obtained from vacuum microwave-dried materials may contain a slightly higher proportion of readily extractable bioactive constituents.

Importantly, phenolic compounds exhibit concentration-dependent biological effects. At moderate concentrations, these compounds often exert protective antioxidant functions, whereas at elevated concentrations they may interact with cellular redox systems and influence mitochondrial metabolism [61]. Such interactions may partially explain the modest reduction in metabolic activity observed at the highest extract concentrations. However, it should be noted that cell viability remained above approximately 80% for both extracts at concentrations up to 200–300 µg/mL, indicating that the extracts were largely non-cytotoxic within this range.

According to widely accepted criteria for in vitro cytotoxicity assessment, compounds that maintain cell viability above 80% are generally considered non-cytotoxic under the tested conditions [62,63]. Therefore, the results suggest that cocoa pod husk extracts exhibit good biocompatibility with RAW264.7 macrophages at moderate concentrations and can be safely applied within this range for subsequent biological investigations, including anti-inflammatory activity assays.

Overall, both Cocoa-A and Cocoa-B extracts demonstrated low cytotoxicity toward macrophage cells and displayed a typical concentration-dependent response at elevated concentrations. The slight difference between Cocoa-A and Cocoa-B may reflect differences in extract composition resulting from the drying process.

**Figure 5:**
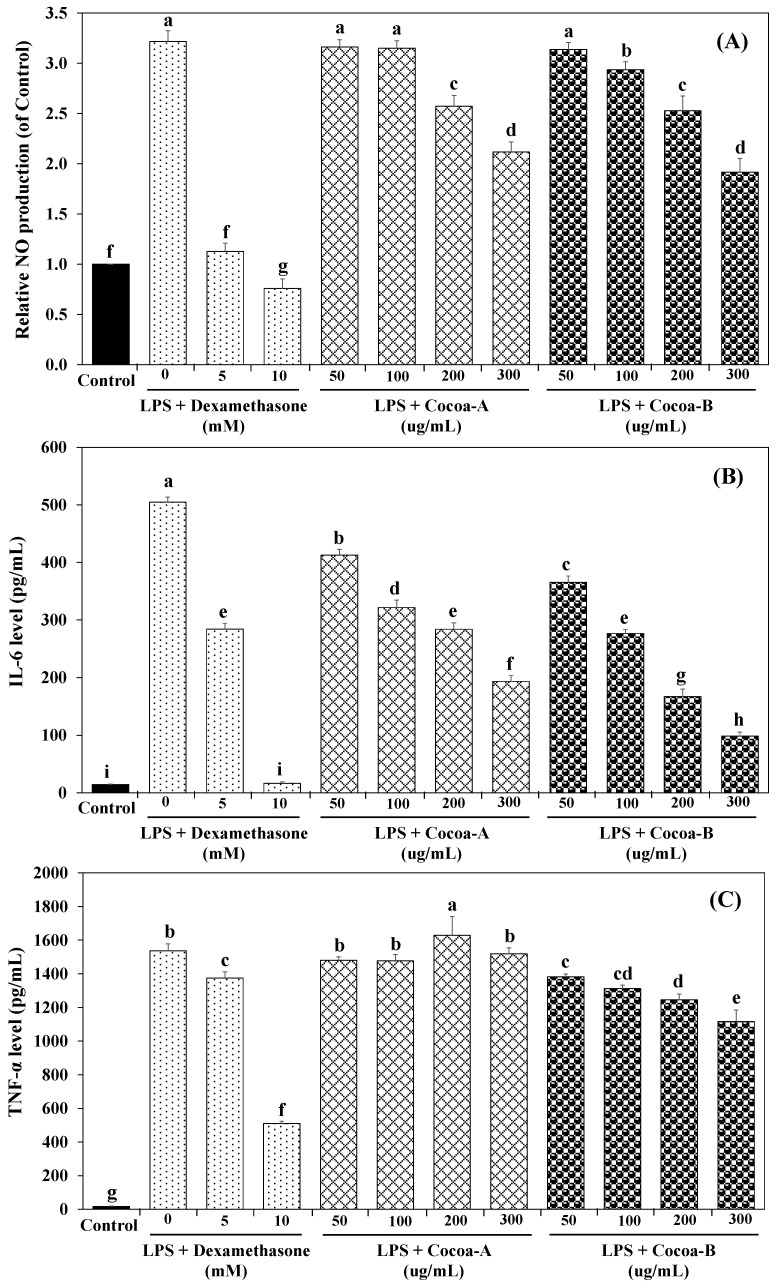
Anti-inflammatory effects of Cocoa-A and Cocoa-B extracts on LPS-stimulated RAW264.7 macrophages. RAW264.7 cells were stimulated with lipopolysaccharide (LPS) and treated with Cocoa-A or Cocoa-B extracts (50, 100, 200, and 300 µg/mL). Dexamethasone (5 and 10 mM) served as the positive control. (**A**) Relative nitric oxide (NO) production expressed as fold change relative to untreated cells. (**B**) Interleukin-6 (IL-6) levels in the culture supernatant. (**C**) Tumor necrosis factor-alpha (TNF-α) levels in the culture supernatant. Different lowercase letters above the bars indicate significant differences among treatments (*p* < 0.05).

Figure 5A–C presents the effects of cocoa pod husk extracts on inflammatory responses in lipopolysaccharide (LPS)-stimulated RAW264.7 macrophages, as determined by the levels of major inflammatory mediators, including nitric oxide (NO), interleukin-6 (IL-6), and tumor necrosis factor-α (TNF-α). LPS stimulation markedly induced an inflammatory response compared with untreated control cells, confirming successful macrophage activation. As shown in Figure 5A, LPS treatment increased NO production to approximately 3.2-fold of the control level. Similarly, pro-inflammatory cytokines were strongly elevated, with IL-6 reaching approximately 500 pg/mL (Figure 5B) and TNF-α approximately 1550 pg/mL (Figure 5C). Treatment with dexamethasone, used as a positive anti-inflammatory control, markedly suppressed the production of these inflammatory mediators, confirming the reliability of the experimental model and the responsiveness of RAW264.7 macrophages to anti-inflammatory agents [64,65].

Treatment with cocoa pod husk extracts significantly attenuated LPS-induced inflammatory mediator production in a concentration-dependent manner. For nitric oxide production (Figure 5A), Cocoa-A gradually reduced NO levels from approximately 3.1-fold at 50–100 µg/mL to around 2.5-fold and 2.1-fold at 200 and 300 µg/mL, respectively. Cocoa-B exhibited a slightly stronger inhibitory effect, decreasing NO production from approximately 3.1-fold at 50 µg/mL to about 1.9-fold at 300 µg/mL. A similar concentration-dependent trend was observed for IL-6 secretion (Figure 5B). Cocoa-A reduced IL-6 levels from approximately 410 pg/mL at 50 µg/mL to about 190 pg/mL at 300 µg/mL. In comparison, Cocoa-B showed a more pronounced inhibitory effect, decreasing IL-6 from approximately 360 pg/mL at 50 µg/mL to nearly 95 pg/mL at 300 µg/mL. In contrast, the suppression of TNF-α production was relatively less pronounced (Figure 5C). Cocoa-A produced minimal reductions in TNF-α levels across the tested concentrations, whereas Cocoa-B showed a clearer decreasing trend, reducing TNF-α secretion from approximately 1380 pg/mL at 50 µg/mL to about 1120 pg/mL at 300 µg/mL. These findings indicate that both extracts can suppress LPS-induced inflammatory responses, with Cocoa-B generally demonstrating stronger inhibitory activity, particularly in the regulation of NO and IL-6 production.

Previous studies have reported that phenolic compounds possess anti-inflammatory properties and may modulate inflammatory signaling pathways in activated macrophages [66]. However, the molecular mechanisms underlying the anti-inflammatory activity of the cocoa pod husk extracts were not investigated in the present study.

The slightly stronger anti-inflammatory activity observed for Cocoa-B may reflect differences in the composition or relative abundance of bioactive compounds resulting from the drying process. However, the present study did not determine which constituents were responsible for the observed differences. In addition, certain thermal processes can promote the formation of smaller phenolic derivatives or enhance the extractability of flavonoids and phenolic acids, which may exhibit stronger biological activity in macrophage systems [67,68]. Although Cocoa-B showed greater suppression of NO and IL-6 than TNF-α, the molecular basis underlying these differences was not investigated in the present study.

Notably, Cocoa-B exhibited stronger anti-inflammatory effects despite Cocoa-A showing higher total phenolic content, while antioxidant capacity measured by DPPH, ABTS, and FRAP assays was comparable between the two extracts (DPPH: 24.94 and 23.77 µmol TEAC/g; ABTS: 33.96 and 34.65 µmol TEAC/g; FRAP: 104.38 and 103.92 µmol TEAC/g for Cocoa-A and Cocoa-B, respectively), as shown in Table 2. These findings suggest that total phenolic content and antioxidant capacity alone may not fully explain the observed anti-inflammatory response. Individual phenolic compounds were quantified by HPLC; however, no correlation analysis was performed between the concentrations of these compounds and the suppression of inflammatory mediators (NO, IL-6, and TNF-α). Consequently, the contribution of individual phenolic compounds to the observed anti-inflammatory activity could not be established. In addition, the underlying molecular mechanisms were not investigated. Further studies integrating phenolic compositional data with bioactivity, together with mechanistic investigations, are required to better elucidate the anti-inflammatory activity of cocoa pod husk extracts.

Collectively, the results demonstrate that cocoa pod husk extracts exhibit anti-inflammatory activity in LPS-stimulated macrophages by suppressing key inflammatory mediators in a concentration-dependent manner. The stronger inhibitory effects observed for Cocoa-B suggest that the drying method applied prior to extraction may influence the anti-inflammatory activity of cocoa pod husk extracts. These findings highlight the potential of cocoa pod husk, an abundant agro-industrial by-product, as a promising source of bioactive compounds for functional food or nutraceutical applications.

Taken together, these findings suggest that the biological activities of cocoa pod husk extracts cannot be explained solely by total phenolic content. Although vacuum microwave drying is generally reported to preserve phenolic compounds through rapid moisture removal, internal heating, and reduced oxidative degradation, these advantages did not directly translate into stronger biological activity in the present study. Vacuum microwave-dried cocoa pod husk extracts generally exhibited slightly higher total phenolic contents and higher concentrations of several phenolic compounds, including *p*-hydroxybenzoic acid, protocatechuic acid, quercetin, and vanillic acid. However, the tray-dried extracts showed substantially higher concentrations of myricetin and epicatechin, which may contribute to their stronger anti-inflammatory activity. These findings suggest that differences in individual phenolic profiles, rather than total phenolic content alone, may influence the observed biological responses. Although antioxidant activity varied among extracts subjected to different drying treatments, its relationship with antibacterial and anti-inflammatory activities was not always directly proportional. Thermal processing may modify the plant matrix, promoting the release of bound phenolic compounds while simultaneously altering phenolic composition, thereby affecting the functional properties of the extracts. Similar observations have been reported for plant-derived materials, where drying and thermal processing influenced phenolic profiles and antioxidant properties depending on the processing conditions and characteristics of the food matrix [69,70].

Nevertheless, the present study did not investigate the individual contributions of specific phenolic compounds or the molecular mechanisms underlying inflammatory mediator suppression and antibacterial activity. Therefore, further studies involving comprehensive phytochemical characterization (e.g., HPLC-MS or LC-MS), correlation analysis between individual compounds and biological activities, and investigation of the molecular mechanisms underlying the observed anti-inflammatory and antibacterial effects are required.

## 4. Conclusions

This study demonstrated that the integration of drying pretreatment and probe ultrasound-assisted extraction is an effective approach for producing bioactive extracts from cocoa pod husk. Among the extraction conditions investigated, aqueous ethanol (1:3, *v*/*v*) was a more suitable solvent than water and absolute ethanol, while a solid-to-solvent ratio of 1:9 (*w*/*v*) provided higher total phenolic content and antioxidant activities than the 1:5 and 1:7 (*w*/*v*) ratios using aqueous ethanol (1:3, *v*/*v*). Probe ultrasonication at 75% amplitude for 15 min yielded the highest total phenolic contents, reaching 5.01 ± 0.14 and 5.41 ± 0.14 mg GAE/g dried cocoa pod husk for the tray-dried extract (Cocoa-B) and vacuum microwave-dried extract (Cocoa-A), respectively. These extracts were subsequently selected for biological evaluation.

The evaluated extracts exhibited antibacterial activity against *Streptococcus mutans*. The selected extracts (Cocoa-A and Cocoa-B) were further evaluated for cellular activities, demonstrating antioxidant activity in HepG2 cells and anti-inflammatory activity through the reduction in intracellular ROS production and suppression of NO, IL-6, and TNF-α production in LPS-stimulated RAW264.7 macrophages. Although Cocoa-A (vacuum microwave-dried extract) contained a higher total phenolic content, Cocoa-B (tray-dried extract) exhibited stronger anti-inflammatory activity, suggesting that the bioactivity of cocoa pod husk extracts was influenced by the drying pretreatment and was not solely determined by total phenolic content. The observed differences may also be associated with variations in phenolic composition; however, the contribution of individual phenolic compounds was not investigated in this study.

These findings provide insights into the effects of drying pretreatment and probe ultrasound-assisted extraction parameters on the recovery of bioactive compounds from cocoa pod husk and support the further development of cocoa pod husk-derived ingredients for functional foods, nutraceuticals, and oral health-related applications. However, the present study did not identify individual phenolic compounds or evaluate the relationships between specific phytochemicals and the observed biological activities. Therefore, the contributions of individual compounds to the antioxidant, antibacterial, and anti-inflammatory effects remain to be clarified. Future studies should include comprehensive chemical profiling and correlation analyses to identify compounds associated with the observed biological activities and to elucidate the mechanisms underlying their antioxidant, antibacterial, and anti-inflammatory effects.

## Figures and Tables

**Table 1:** Types and contents of phenolic compounds in tray-dried cocoa pod husk and its extracts obtained using aqueous ethanol (1:3 *v*/*v*) and ethanol at different solid to solvent ratios (1:5–1:9 *w*/*v*) with an amplitude of 50% and an extraction time of 30 min.

Types of Phenolic Compounds	Contents of Phenolic Compounds (µg/g Dried Cocoa Pod Husk)				
	Tray-Dried Cocoa Pod Husk	Tray-Dried Cocoa Pod Husk Extract (Aqueous Ethanol, 1:3 *v*/*v*; Solid to Solvent Ratio 1:5 *w*/*v*)	Tray-Dried Cocoa Pod Husk Extract (Aqueous Ethanol, 1:3 *v*/*v*; Solid to Solvent Ratio 1:7 *w*/*v*)	Tray-Dried Cocoa Pod Husk Extract (Aqueous Ethanol, 1:3 *v*/*v*; Solid to Solvent Ratio 1:9 *w*/*v*)	Tray-Dried Cocoa Pod Husk Extract (Ethanol; Solid to Solvent Ratio 1:7 *w*/*v*)
Gallic acid	0.00	0.00 ± 0.00	0.00 ± 0.00	0.00 ± 0.00	0.00 ± 0.00
Theobromine	0.00	2.66 ± 0.02 ^b^	2.81 ± 0.15 ^b^	4.18 ± 0.16 ^a^	0.92 ± 0.01 ^c^
Protocatechuic acid	0.00	2.49 ± 0.06 ^c^	3.28 ± 0.04 ^b^	5.25 ± 0.13 ^a^	0.00 ± 0.00
*p*-Hydroxybenzoic acid	0.00	50.30 ± 0.01 ^c^	55.48 ± 0.24 ^b^	95.31 ± 0.71 ^a^	0.00 ± 0.00
Catechin	0.00	0.00 ± 0.00	0.00 ± 0.00	0.00 ± 0.00	0.00 ± 0.00
Chlorogenic acid	0.00	31.10 ± 0.53 ^c^	34.52 ± 0.66 ^b^	55.07 ± 0.13 ^a^	0.00 ± 0.00
Caffeine	0.00	0.19 ± 0.01 ^b^	0.23 ± 0.02 ^b^	0.37 ± 0.02 ^a^	0.00 ± 0.00
Vanillic acid	0.00	3.98 ± 0.17 ^c^	4.45 ± 0.12 ^b^	6.93 ± 0.12 ^a^	0.00 ± 0.00
Caffeic acid	0.00	0.00 ± 0.00	0.00 ± 0.00	0.00 ± 0.00	0.00 ± 0.00
Syringic acid	0.00	32.29 ± 0.56 ^c^	36.80 ± 0.01 ^b^	62.71 ± 0.24 ^a^	0.00 ± 0.00
Epicatechin	18.76 ± 0.62 ^e^	201.62 ± 0.03 ^c^	231.92 ± 1.13 ^b^	381.62 ± 1.30 ^a^	87.41 ± 0.21 ^d^
Vanillin	0.00	0.00 ± 0.00	0.00 ± 0.00	0.00 ± 0.00	0.00 ± 0.00
*p*-Coumaric acid	0.00	0.00 ± 0.00	0.00 ± 0.00	0.00 ± 0.00	0.00 ± 0.00
Ferulic acid	0.00	37.11 ± 0.10 ^c^	41.41 ± 0.27 ^b^	71.89 ± 0.20 ^a^	14.64 ± 0.08 ^d^
Sinapic acid	0.00	0.00 ± 0.00	0.00 ± 0.00	0.00 ± 0.00	0.00 ± 0.00
Rutin	0.00	24.00 ± 0.61 ^c^	27.62 ± 0.44 ^b^	45.94 ± 0.74 ^a^	7.06 ± 0.18 ^d^
Myricetin	0.00	0.00 ± 0.00	0.00 ± 0.00	0.00 ± 0.00	0.00 ± 0.00
Quercetin	0.00	0.00 ± 0.00	0.00 ± 0.00	0.00 ± 0.00	0.00 ± 0.00
Trans-cinnamic acid	0.00	0.00 ± 0.00	0.00 ± 0.00	0.00 ± 0.00	0.00 ± 0.00

Values are expressed as mean ± SD. Different superscript letters within the same row indicate significant differences (*p* < 0.05).

**Table 2:** Total phenolic content and antioxidant activity of dried cocoa pod husk extracts obtained by probe ultrasonication using aqueous ethanol (1:3 *v*/*v*) at a solid to solvent ratio of 1:9 (*w*/*v*).

Sample	Probe Ultrasonication Extraction Conditions		Total Phenolic Content (mg GAE/g Dried Cocoa Pod Husk)	Antioxidant Activity (µmol TEAC/g Dried Cocoa Pod Husk)		
	Time (min)	Amplitude (%)		DPPH	ABTS	FRAP
**Tray-dried cocoa pod husk**	15	25	4.06 ± 0.08 ^g^	26.17 ± 0.18 ^e^	38.28 ± 1.37 ^ab^	89.13 ± 0.17 ^j^
		50	4.23 ± 0.04 ^fg^	25.08 ± 0.03 ^h^	35.72 ± 0.09 ^de^	103.33 ± 3.37 ^ef^
		75	5.01 ± 0.14 ^cd^	23.77 ± 0.07 ^i^	34.65 ± 1.23 ^ef^	103.92 ± 0.48 ^ef^
	30	25	4.09 ± 0.37 ^g^	25.76 ± 0.11 ^g^	37.65 ± 0.15 ^abc^	91.40 ± 0.05 ^ij^
		50	5.00 ± 0.05 ^cd^	23.83 ± 0.13 ^i^	36.08 ± 0.90 ^cde^	100.77 ± 1.20 ^efg^
		75	4.90 ± 0.08 ^cd^	20.13 ± 0.18 ^l^	35.81 ± 1.13 ^de^	114.04 ± 1.59 ^bc^
	45	25	4.27 ± 0.24 ^f^	26.62 ± 0.24 ^c^	37.53 ± 0.99 ^abc^	97.60 ± 0.85 ^gh^
		50	4.88 ± 0.04 ^d^	22.57 ± 0.02 ^j^	35.05 ± 1.58 ^ef^	108.79 ± 0.12 ^d^
		75	4.22 ± 0.03 ^fg^	15.64 ± 0.14 ^n^	34.52 ± 0.69 ^ef^	103.71 ± 1.07 ^ef^
**Vacuum microwave-dried cocoa pod husk**	15	25	3.75 ± 0.13 ^h^	27.83 ± 0.15 ^b^	29.20 ± 0.94 ^hi^	100.52 ± 1.02 ^efg^
		50	5.23 ± 0.00 ^b^	26.67 ± 0.17 ^c^	36.03 ± 0.06 ^cde^	112.00 ± 0.19 ^cd^
		75	5.41 ± 0.14 ^a^	24.94 ± 0.05 ^h^	33.96 ± 0.03 ^f^	104.38 ± 0.19 ^e^
	30	25	3.85 ± 00.07 ^h^	28.01 ± 0.06 ^a^	29.51 ± 2.50 ^h^	100.01 ± 0.13 ^fg^
		50	5.07 ± 0.10 ^bc^	26.45 ± 0.18 ^d^	38.65 ± 2.05 ^a^	115.82 ± 1.91 ^b^
		75	4.63 ± 0.12 ^e^	21.13 ± 0.14 ^k^	34.68 ± 0.57 ^ef^	122.35 ± 9.04 ^a^
	45	25	3.77 ± 0.23 ^h^	25.95 ± 0.02 ^f^	27.76 ± 0.51 ^i^	94.73 ± 0.15 ^hi^
		50	5.21 ± 0.14 ^b^	23.67 ± 0.08 ^i^	36.78 ± 3.08 ^bcd^	123.99 ± 8.06 ^a^
		75	4.57 ± 0.02 ^e^	16.46 ± 0.01 ^m^	31.61 ± 0.43 ^g^	116.65 ± 0.19 ^b^

Values are expressed as mean ± SD. Different superscript letters within the same row indicate significant differences (*p* < 0.05).

**Table 3:** Types and contents of phenolic compounds in extracts from tray-dried cocoa pod husk obtained using probe ultrasonication with aqueous ethanol (1:3 *v*/*v*) at a solid to solvent ratio of 1:9 (*w*/*v*).

Types of Phenolic Compounds	Phenolic Content of Tray-Dried Cocoa Pod Husk Extracts (µg/g Dried Cocoa Pod Husk)								
	TD-A25-T15	TD-A50-T15	TD-A75-T15	TD-A25-T30	TD-A50-T30	TD-A75-T30	TD-A25-T45	TD-A50-T45	TD-A75-T45
Theobromine	2.53 ± 0.04 ^cd^	3.34 ± 0.11 ^ab^	3.40 ± 0.13 ^ab^	2.38 ± 0.09 ^d^	3.44 ± 0.20 ^ab^	3.36 ± 0.12 ^ab^	2.73 ± 0.18 ^c^	3.14 ± 0.27 ^b^	3.54 ± 0.43 ^a^
Protocatechuic acid	9.12 ± 0.31 ^d^	14.71 ± 0.31 ^a^	15.24 ± 0.87 ^a^	9.02 ± 0.47 ^d^	14.65 ± 0.32 ^a^	15.33 ± 0.60 ^a^	10.30 ± 0.57 ^c^	12.96 ± 1.01 ^b^	13.79 ± 0.30 ^b^
*p*-Hydroxybenzoic acid	162.78 ± 5.40 ^d^	308.55 ± 5.32 ^b^	317.97 ± 8.66 ^b^	175.26 ± 5.13 ^d^	328.97 ± 3.13 ^b^	351.34 ± 20.75 ^a^	221.73 ± 5.95 ^c^	308.28 ± 33.11 ^b^	327.71 ± 11.10 ^b^
Vanillic acid	16.97 ± 2.73 ^de^	22.98 ± 2.15 ^a^	22.05 ± 0.62 ^ab^	15.74 ± 0.57 ^e^	21.76 ± 1.07 ^ab^	21.85 ± 1.83 ^ab^	18.65 ± 0.40 ^cd^	20.45 ± 1.42 ^bc^	21.16 ± 0.46 ^ab^
Syringic acid	18.07 ± 3.24 ^g^	39.33 ± 3.25 ^e^	42.62 ± 1.27 ^b^	24.08 ± 0.77 ^f^	42.94 ± 1.07 ^b^	46.31 ± 2.31 ^a^	28.73 ± 1.76 ^e^	32.87 ± 2.68 ^d^	35.96 ± 1.34 ^d^
Epicatechin	282.53 ± 3.76 ^f^	503.41 ± 18.05 ^b^	536.65 ± 17.54 ^a^	310.60 ± 12.36 ^e^	538.90 ± 18.11 ^a^	560.54 ± 20.86 ^a^	383.08 ± 1.76 ^d^	465.66 ± 40.00 ^c^	494.88 ± 4.58 ^b^
*p*-Coumaric acid	5.35 ± 0.37 ^f^	20.83 ± 4.55 ^cd^	24.09 ± 0.92 ^b^	12.71 ± 0.53 ^e^	23.32 ± 1.04 ^bc^	27.83 ± 1.68 ^a^	15.03 ± 0.36 ^e^	19.09 ± 1.82 ^d^	21.78 ± 1.13 ^bcd^
Ferulic acid	108.28 ± 31.45 ^e^	255.83 ± 8.18 ^b^	275.07 ± 9.97 ^b^	169.93 ± 8.54 ^d^	276.16 ± 2.40 ^b^	336.38 ± 24.49 ^a^	197.35 ± 3.82 ^c^	263.54 ± 28.29 ^b^	312.41 ± 3.93 ^a^
Rutin	15.30 ± 1.81 ^e^	30.67 ± 1.13 ^ab^	31.48 ± 1.16 ^ab^	19.93 ± 0.40 ^d^	31.00 ± 0.63 ^ab^	33.19 ± 3.13 ^a^	22.57 ± 0.13 ^c^	29.46 ± 2.43 ^b^	32.11 ± 0.94 ^a^
Myricetin	4453.54 ± 665.55 ^ab^	4131.46 ± 139.04 ^b^	4077.18 ± 154.69 ^b^	4867.42 ± 120.51 ^a^	4028.93 ± 213.93 ^b^	4388.87 ± 260.61 ^ab^	4296.94 ± 156.03 ^b^	3929.17 ± 392.29 ^b^	3878.58 ± 604.63 ^b^
Trans-cinnamic acid	38.10 ± 4.63 ^ab^	35.27 ± 4.53 ^ab^	31.69 ± 3.30 ^b^	41.07 ± 5.34 ^a^	36.44 ± 2.22 ^ab^	35.81 ± 2.82 ^ab^	42.78 ± 5.21 ^a^	35.39 ± 4.59 ^ab^	41.16 ± 3.20 ^a^

Values are expressed as mean ± SD. Different superscript letters within the same row indicate significant differences (*p* < 0.05). TD denotes tray-dried cocoa pod husk. A denotes probe ultrasonication amplitude (%). T denotes extraction time (min). The numerical values following A and T represent the ultrasonication amplitude (%) and extraction time (min), respectively.

**Table 4:** Types and contents of phenolic compounds in extracts from vacuum microwave-dried cocoa pod husk obtained using probe ultrasonication with aqueous ethanol (1:3 *v*/*v*) at a solid to solvent ratio of 1:9 (*w*/*v*).

Types of Phenolic Compounds	Phenolic Content of Vacuum Microwave-Dried Cocoa Pod Husk Extracts (µg/g Dried Cocoa Pod Husk)								
	MD-A25-T15	MD-A50-T15	MD-A75-T15	MD-A25-T30	MD-A50-T30	MD-A75-T30	MD-A25-T45	MD-A50-T45	MD-A75-T45
Theobromine	5.35 ± 0.47 ^b^	9.15 ± 0.72 ^a^	9.53 ± 0.49 ^a^	6.22 ± 0.83 ^b^	5.93 ± 4.42 ^b^	1.53 ± 0.17 ^c^	1.25 ± 0.04 ^c^	1.54 ± 0.04 ^c^	1.47 ± 0.05 ^c^
Protocatechuic acid	15.37 ± 2.75 ^de^	33.34 ± 0.49 ^ab^	36.63 ± 2.33 ^a^	20.65 ± 1.57 ^cd^	26.47 ± 15.67 ^bc^	9.57 ± 0.58 ^ef^	5.57 ± 0.28 ^f^	9.45 ± 0.16 ^ef^	8.61 ± 0.62 ^ef^
*p*-Hydroxybenzoic acid	182.87 ± 13.98 ^f^	367.04 ± 12.10 ^c^	403.70 ± 17.26 ^b^	217.34 ± 21.85 ^e^	432.00 ± 13.14 ^a^	323.24 ± 28.83 ^d^	213.19 ± 2.84 ^e^	361.25 ± 16.84 ^c^	333.31 ± 12.39 ^d^
Vanillic acid	25.86 ± 1.70 ^f^	39.29 ± 1.12 ^ab^	39.26 ± 2.20 ^ab^	28.88 ± 2.36 ^e^	41.13 ± 1.51 ^a^	34.03 ± 3.72 ^c^	30.30 ± 2.41 ^de^	37.33 ± 1.37 ^b^	33.15 ± 0.71 ^cd^
Syringic acid	9.00 ± 0.31 ^e^	17.28 ± 0.43 ^bc^	18.56 ± 0.59 ^bc^	9.95 ± 1.07 ^de^	21.28 ± 2.70 ^a^	16.65 ± 1.66 ^c^	11.50 ± 0.26 ^d^	18.84 ± 1.53 ^b^	18.16 ± 0.36 ^bc^
Epicatechin	251.16 ± 22.29 ^e^	462.26 ± 5.86 ^bc^	480.85 ± 18.25 ^bc^	283.73 ± 29.27 ^de^	573.12 ± 62.57 ^a^	454.16 ± 39.63 ^c^	325.13 ± 5.52 ^d^	504.37 ± 29.10 ^b^	464.10 ± 8.82 ^bc^
*p*-Coumaric acid	6.40 ± 0.59 ^d^	15.57 ± 0.83 ^b^	15.86 ± 0.63 ^b^	8.48 ± 1.02 ^cd^	19.50 ± 2.65 ^a^	15.49 ± 2.74 ^b^	9.88 ± 0.39 ^c^	17.17 ± 1.39 ^b^	16.93 ± 0.43 ^b^
Ferulic acid	71.17 ± 2.50 ^d^	135.53 ± 7.88 ^c^	145.94 ± 3.38 ^bc^	75.34 ± 4.18 ^d^	175.24 ± 23.79 ^a^	137.52 ± 23.34 ^c^	86.80 ± 5.72 ^d^	159.93 ± 8.92 ^ab^	163.58 ± 6.56 ^ab^
Rutin	12.44 ± 1.35 ^e^	24.82 ± 0.61 ^bc^	26.68 ± 1.66 ^b^	15.80 ± 1.60 ^d^	30.69 ± 1.09 ^a^	23.35 ± 1.73 ^c^	16.46 ± 0.45 ^d^	26.67 ± 1.03 ^b^	26.25 ± 0.59 ^b^
Myricetin	2029.75 ± 84.30 ^c^	2538.63 ± 112.05 ^b^	2538.85 ± 50.02 ^b^	1899.22 ± 75.53 ^c^	3226.66 ± 702.88 ^a^	2453.01 ± 186.79 ^b^	1983.93 ± 59.16 ^c^	2561.66 ± 44.03 ^b^	2436.34 ± 110.08 ^b^
Quercetin	299.73 ± 23.04 ^a^	280.78 ± 38.06 ^a^	286.48 ± 29.13 ^a^	184.93 ± 75.19 ^b^	262.13 ± 63.25 ^a^	232.30 ± 28.04 ^ab^	171.61 ± 27.80 ^b^	258.01 ± 38.75 ^a^	186.83 ± 8.05 ^b^
Trans-cinnamic acid	24.41 ± 2.51 ^de^	39.67 ± 5.09 ^ab^	43.23 ± 0.72 ^a^	32.98 ± 7.09 ^bc^	24.42 ± 5.45 ^de^	22.29 ± 4.06 ^de^	18.23 ± 3.28 ^e^	26.41 ± 7.16 ^cd^	27.83 ± 2.69 ^cd^

Values are expressed as mean ± SD. Different superscript letters within the same row indicate significant differences (*p* < 0.05). MD denotes vacuum microwave-dried cocoa pod husk. A denotes probe ultrasonication amplitude (%). T denotes extraction time (min). The numerical values following A and T represent the ultrasonication amplitude (%) and extraction time (min), respectively.

## Data Availability

The data will be available on request.

## References

[1] Anoraga S.B., Shamsudin R., Hamzah M.H., Sharif S., Saputra A.D. (2024). Cocoa by-products: A comprehensive review on potential uses, waste management, and emerging green technologies for cocoa pod husk utilization. Heliyon.

[2] Belwal T., Cravotto C., Ramola S., Thakur M., Chemat F., Cravotto G. (2022). Bioactive compounds from cocoa Husk: Extraction, analysis and applications in food production chain. Foods.

[3] de Souza Vandenberghe L.P., Valladares-Diestra K.K., Bittencourt G.A., de Mello A.F.M., Vasquez Z.S., de Oliveira P.Z., de Melo Pereira G.V., Soccol C.R. (2022). Added-value biomolecules’ production from cocoa pod husks: A review. Bioresour. Technol..

[4] Nguyen V.L., Tran M.T., Nguyen-Thi T.D., Nguyen M.A., Le M.T., Nguyen T.M., Nguyen Q.D. (2025). Valorization of cocoa (*Theobroma cacao* L.) pod husks as a fruit pulp substitute in mango jam formulations: Effects on jam qualities during storage and sensory discrimination. Sustain. Food Technol..

[5] Botella-Martínez C., Lucas-Gonzalez R., Ballester-Costa C., Pérez-Álvarez J.A., Fernández-López J., Delgado-Ospina J., Chaves-López C., Viuda-Martos M. (2021). Ghanaian cocoa (*Theobroma cacao* L.) bean shells coproducts: Effect of particle size on chemical composition, bioactive compound content and antioxidant activity. Agronomy.

[6] Barbosa-Pereira L., Belviso S., Ferrocino I., Rojo-Poveda O., Zeppa G. (2021). Characterization and classification of cocoa bean shells from different regions of venezuela using HPLC-PDA-MS/MS and spectrophotometric techniques coupled to chemometric analysis. Foods.

[7] Tušek K., Valinger D., Jurina T., Sokač Cvetnić T., Gajdoš Kljusurić J., Benković M. (2024). Bioactives in cocoa: Novel findings, health benefits, and extraction techniques. Separations.

[8] Fideles S.O.M., Ortiz A.d.C., Reis C.H.B., Buchaim D.V., Buchaim R.L. (2023). Biological properties and antimicrobial potential of cocoa and its effects on systemic and oral health. Nutrients.

[9] Quiceno-Suarez A., Cadena-Chamorro E.M., Ciro-Velásquez H.J. (2025). Processing of agroindustrial byproducts from the cocoa (*Theobroma Cacao* L.) production chain to obtain bioactive compounds through supercritical fluid extraction and enzyme-assisted extraction and their potential application. J. Chem. Technol. Biotechnol..

[10] Thamkaew G., Sjöholm I., Galindo F.G. (2021). A review of drying methods for improving the quality of dried herbs. Crit. Rev. Food Sci. Nutr..

[11] Nakra S., Tripathy S., Srivastav P.P. (2025). Drying as a preservation strategy for medicinal plants: Physicochemical and functional outcomes for food and human health. Phytomed. Plus.

[12] ElGamal R., Song C., Rayan A.M., Liu C., Al-Rejaie S., ElMasry G. (2023). Thermal degradation of bioactive compounds during drying process of horticultural and agronomic products: A comprehensive overview. Agronomy.

[13] Santos A.A.d.L., Leal G.F., Marques M.R., Reis L.C.C., Junqueira J.R.d.J., Macedo L.L., Corrêa J.L.G. (2025). Emerging drying technologies and their impact on bioactive compounds: A systematic and bibliometric review. Appl. Sci..

[14] Guo W., Wang M., Shen L., Liu C., Liu C., Zheng X. (2025). Effects of microwave vacuum drying on drying characteristics, bioactive components, antioxidant capacity, and starch structure of germinated red adzuki beans. Food Chem. X.

[15] Dehghannya J., Habibi-Ghods M. (2025). Microwave drying of food materials: Principles, hybrid techniques, modeling approaches, and emerging innovations. Compr. Rev. Food Sci. Food Saf..

[16] Huang Y., Sun Y., Mehmood A., Lu T., Chen X. (2024). Unraveling the temporal changes of maillard reaction products and aroma profile in coffee leaves during hot-air drying. J. Food Compos. Anal..

[17] Kumar C., Karim M.A. (2019). Microwave-convective drying of food materials: A critical review. Crit. Rev. Food Sci. Nutr..

[18] Bhadange Y.A., Carpenter J., Saharan V.K. (2024). A comprehensive review on advanced extraction techniques for retrieving bioactive components from natural sources. ACS Omega.

[19] Sun S., Yu Y., Jo Y., Han J.H., Xue Y., Cho M., Bae S.-J., Ryu D., Park W., Ha K.-T. (2025). Impact of extraction techniques on phytochemical composition and bioactivity of natural product mixtures. Front. Pharmacol..

[20] Shen L., Pang S., Zhong M., Sun Y., Qayum A., Liu Y., Rashid A., Xu B., Liang Q., Ma H. (2023). A comprehensive review of ultrasonic assisted extraction (UAE) for bioactive components: Principles, advantages, equipment, and combined technologies. Ultrason. Sonochem..

[21] Islam M., Malakar S., Rao M.V., Kumar N., Sahu J.K. (2023). Recent advancement in ultrasound-assisted novel technologies for the extraction of bioactive compounds from herbal plants: A review. Food Sci. Biotechnol..

[22] Carreira-Casais A., Otero P., Garcia-Perez P., Garcia-Oliveira P., Pereira A.G., Carpena M., Soria-Lopez A., Simal-Gandara J., Prieto M.A. (2021). Benefits and drawbacks of ultrasound-assisted extraction for the recovery of bioactive compounds from marine algae. Int. J. Environ. Res. Public Health.

[23] Dranca F., Oroian M. (2015). Total monomeric anthocyanin, total phenolic content and antioxidant activity of extracts from eggplant (*Solanum Melongena* L.) peel using ultrasonic treatments. J. Food Process. Eng..

[24] Ferarsa S., Zhang W., Moulai-Mostefa N., Ding L., Jaffrin M.Y., Grimi N. (2018). Recovery of anthocyanins and other phenolic compounds from purple eggplant peels and pulps using ultrasonic-assisted extraction. Food Bioprod. Process..

[25] Liaudanskas M., Viškelis P., Raudonis R., Kviklys D., Uselis N., Janulis V. (2014). Phenolic composition and antioxidant activity of Malus domestica leaves. Sci. World J..

[26] Benítez-Correa E., Bastías-Montes J.M., Acuña-Nelson S., Muñoz-Fariña O. (2023). Effect of choline chloride-based deep eutectic solvents on polyphenols extraction from cocoa (*Theobroma cacao* L.) bean shells and antioxidant activity of extracts. Curr. Res. Food Sci..

[27] Brand-Williams W., Cuvelier M.E., Berset C. (1995). Use of a free radical method to evaluate antioxidant activity. LWT—Food Sci. Technol..

[28] Chen H.-Y., Lin Y.-C., Hsieh C.-L. (2007). Evaluation of antioxidant activity of aqueous extract of some selected nutraceutical herbs. Food Chem..

[29] Benzie I.F.F., Strain J.J. (1996). The ferric reducing ability of plasma (FRAP) as a measure of “antioxidant power”: The FRAP assay. Anal. Biochem..

[30] Szydłowska-Czerniak A., Dianoczki C., Recseg K., Karlovits G., Szłyk E. (2008). Determination of antioxidant capacities of vegetable oils by ferric-ion spectrophotometric methods. Talanta.

[31] Barrios-Rodríguez Y.F., Salas-Calderón K.T., Orozco-Blanco D.A., Gentile P., Girón-Hernández J. (2022). Cocoa pod husk: A high-pectin source with applications in the food and biomedical fields. ChemBioEng Rev..

[32] Santiago-Gómez I., Carrera-Lanestosa A., González-Alejo F.A., Guerra-Que Z., García-Alamilla R., Rivera-Armenta J.L., García-Alamilla P. (2025). Pectin Extraction Process from Cocoa Pod Husk (Theobroma cacao L.) and Characterization by Fourier Transform Infrared Spectroscopy. ChemEngineering.

[33] Hamed M., Abdallah I.A., Bedair A., Mansour F.A. (2023). Sample preparation methods for determination of quercetin and quercetin glycosides in diverse matrices. Microchem. J..

[34] Zhu X., Ding G., Ren S., Xi J., Liu K. (2024). The bioavailability, absorption, metabolism, and regulation of glucolipid metabolism disorders by quercetin and its important glycosides: A review. Food Chem..

[35] Cho J., Kim K., Lee J.G. (2025). Quercetin in food: Structure, biosynthesis, toxicity, analytical method, occurrence and risk assessments. Appl. Biol. Chem..

[36] Carvalho G.R., Monteiro R.L., Laurindo J.B., August P.E.D. (2021). Microwave and microwave-vacuum drying as alternatives to convective drying in barley malt processing. Innov. Food Sci. Emerg. Technol..

[37] Gong S., Niu Y., Yuwen C., Liu B. (2025). Microwave drying of tricholoma matsutake: Dielectric properties, mechanism, and process optimization. Foods.

[38] Hu Q., He Y., Wang F., Wu J., Ci Z., Chen L., Xu R., Yang M., Lin J., Han L. (2021). Microwave technology: A novel approach to the transformation of natural metabolites. Chin. Med..

[39] de Andrade Bulos R.B., de Souza C.O., de Quadros C.P., Leme O.A.D., Corrêa L.C., Oliveira M.B.P.P., Machado S., Biasoto A.C.T., Tavares P.P.L.G., Nascimento R.Q. (2025). Impact of drying and storage conditions on the bioactive and nutritional properties of malolactic wine lees. Foods.

[40] Ahmad N., Zuo Y., Anwar F., Abbas A., Shahid M., Hassan A., Bilal M., Rasheed T. (2021). Ultrasonic-assisted extraction as a green route for hydrolysis of bound phenolics in selected wild fruits: Detection and systematic characterization using GC–MS–TIC method. Process Biochem..

[41] Saad A.M., Mohammed D.M., Alkafaas S.S., Ghosh S., Negm S.H., Salem H.M., Fahmy M.A., Semary H.E., Ibrahim E.H., AbuQamar S.F. (2025). Dietary polyphenols and human health: Sources, biological activities, nutritional and immunological aspects, and bioavailability—A comprehensive review. Front. Immunol..

[42] Chen B., Wan Y., Lu Z., Li H., Liu S., Jia G., Huang D. (2026). Comparative drying of pomelo peel and optimization using ultrasound-assisted near-infrared drying. Ultrason. Sonochem..

[43] Liu Z., Wu H., Holland B., Barrow C.J., Suleria H.A.R. (2024). An optimization of the extraction of phenolic compounds from grape marc: A comparison between conventional and ultrasound assisted methods. Chemosensors.

[44] Expósito-Almellón X., Munguía-Ubierna A., Duque-Soto C., Borrás-Linares I., Quirantes-Piné R., Lozano-Sánchez J. (2025). Optimized ultrasound-assisted extraction for enhanced recovery of valuable phenolic compounds from olive by-products. Antioxidants.

[45] Demesa A.G., Saavala S., Pöysä M., Koiranen T. (2024). Overview and toxicity assessment of ultrasound-assisted extraction of natural ingredients from plants. Foods.

[46] Shehzadi F., Shoaib M., Munir S., Abdi G. (2026). Ultrasound-assisted extraction of bioactive compounds from pomegranate peel and seed: A comprehensive review of key parameters and optimization strategies. Ultrason. Sonochem..

[47] Tao Y., Zhang Z., Sun D.-W. (2014). Kinetic modeling of ultrasound-assisted extraction of phenolic compounds from grape marc: Influence of acoustic energy density and temperature. Ultrason. Sonochem..

[48] Prempeh N.Y.A., Nunekpeku X., Murugesan A., Li H. (2025). Ultrasound in the food industry: Mechanisms and applications for non-invasive texture and quality analysis. Foods.

[49] Rybak K., Skarzyńska A., Ossowski S., Dadan M., Pobiega K., Nowacka M. (2025). Insight into the molecular and structural changes in red pepper induced by direct and indirect ultrasonic treatments. Molecules.

[50] Nguyen V.T., Nguyen N.H. (2017). Proximate composition, extraction, and purification of theobromine from cacao pod husk (*Theobroma cacao* L.). Technologies.

[51] Yang X., Ma Z., Wan F., Chen A., Zhang W., Xu Y., Zang Z., Huang X. (2024). Different pretreatment methods to strengthen the microwave vacuum drying of honeysuckle: Effects on the moisture migration and physicochemical quality. Foods.

[52] Snoussi A., Essaidi I., Koubaier H.B.H., Zrelli H., Alsafari I., Živoslav T., Mihailovic J., Khan M., Omri A.E., Veličković T.C. (2021). Drying methodology effect on phenolic content and antioxidant activity of Myrtus communis leaves extracts. BMC Chem..

[53] Hu D., Liu X., Qin Y., Yan J., Li R., Yang Q. (2023). The impact of different drying methods on the physical properties, bioactive components, antioxidant capacity, volatile components and industrial application of coffee peel. Food Chem. X.

[54] Griendling K.K., Touyz R.M., Zweier J.L., Dikalov S., Chilian W., Chen Y.R., Harrison D.G., Bhatnagar A. (2016). Measurement of reactive oxygen species, reactive nitrogen species, and redox-dependent signaling in the cardiovascular system. Circ. Res..

[55] Kurutas E.B. (2015). The importance of antioxidants which play the role in cellular response against oxidative/nitrosative stress: Current state. Nutr. J..

[56] Chriscensia E., Nathanael J., Perwitasari U., Putra A.B.N., Adiyanto S.A., Hartrianti P. (2024). Potential utilisation of theobroma cacao pod husk extract: Protective capability evaluation against pollution models and formulation into niosomes. Trop. Life Sci. Res..

[57] Anatachodwanit A., Chanpirom S., Tree-Udom T., Kitthaweesinpoon S., Jiamphun S., Aryuwat O., Tantapakul C., Vinardell M.P., Sripisut T. (2025). Upcycled cocoa pod husk: A sustainable source of phenol and polyphenol ingredients for skin hydration, whitening, and anti-aging. Life.

[58] Martínez R., Torres P., Meneses M.A., Figueroa J.G., Pérez-Álvarez J.A., Viuda-Martos M. (2012). Chemical, technological and in vitro antioxidant properties of cocoa (*Theobroma cacao* L.) co-products. Food Res. Int..

[59] Ghasemi M., Turnbull T., Sebastian S., Kempson I. (2021). The MTT assay: Utility, limitations, pitfalls, and interpretation in bulk and single-cell analysis. Int. J. Mol. Sci..

[60] Hao M., Yu Z., Niu L., Han L., Gu M., Xu Y., Zhang Z., Zhou C., Li D., Dai Z. (2025). Effects of different drying methods on the quality, nutritional components, and in vitro bioaccessibility of kiwifruit. Food Sci. Nutr..

[61] Ahmad Z., Rauf A., Orhan I.E., Mubarak M.S., Akram Z., Islam M.R., Imran M., Edis Z., Kondapavuluri B.K., Thangavelu L. (2025). Antioxidant potential of polyphenolic compounds, sources, extraction, purification and characterization techniques: A focused review. Food Sci. Nutr..

[62] Gruber S., Nickel A. (2023). Toxic or not toxic? The specifications of the standard ISO 10993-5 are not explicit enough to yield comparable results in the cytotoxicity assessment of an identical medical device. Front. Med. Technol..

[63] (2009). Biological Evaluation of Medical Devices—Part 5: Tests for In Vitro Cytotoxicity.

[64] Jeong Y.H., Oh Y.C., Cho W.K., Shin H., Lee K.Y., Ma J.Y. (2016). Anti-inflammatory effects of Viola yedoensis and the application of cell extraction methods for investigating bioactive constituents in macrophages. BMC Complement. Altern. Med..

[65] Srisai P., Suriyaprom S., Khacha-Ananda S., Panya A., Bäumler H., Tragoolpua Y. (2025). Inhibition of free radicals and inflammation on RAW264.7 macrophage cell line by Arthrospira platensis extract. Sci. Rep..

[66] Sahakyan N. (2026). Plant-derived phenolics as regulators of nitric oxide production in microglia: Mechanisms and therapeutic potential. Med. Gas Res..

[67] Liu X., Yan X., Bi J., Wu X., Liu J., Zhou M. (2020). Identification of phenolic compounds and antioxidant activity of guava dehydrated by different drying methods. Dry. Technol..

[68] Zhang Y., Liu H. (2022). Editorial: Chemical and biological changes of polyphenols caused by food thermal processing. Front. Nutr..

[69] Wang Z., Li S., Ge S., Lin S. (2020). Review of distribution, extraction methods, and health benefits of bound phenolics in food plants. J. Agric. Food Chem..

[70] Wu Y., Liu Y., Jia Y., Feng C.-H., Zhang H., Ren F., Zhao G. (2024). Effects of thermal processing on natural antioxidants in fruits and vegetables. Food Res. Int..

